# Copy number variation and elevated genetic diversity at immune trait loci in Atlantic and Pacific herring

**DOI:** 10.1186/s12864-024-10380-5

**Published:** 2024-05-10

**Authors:** Fahime Mohamadnejad Sangdehi, Minal S. Jamsandekar, Erik D. Enbody, Mats E. Pettersson, Leif Andersson

**Affiliations:** 1 Department of Medical Biochemistry and Microbiology, Uppsala University, Uppsala, Sweden; 2 Department of Veterinary Integrative Biosciences, Texas A&M University, College Station, USA; 3 Present Address: Department of Molecular and Human Genetics, Baylor College of Medicine, Houston, TX USA; 4 Present Address: Department of Biomolecular Engineering, University of California, Santa Cruz, USA

**Keywords:** Comparative genomics, Genetic diversity, Copy number variation, Immune gene clusters, Atlantic herring, PacBio long reads

## Abstract

**Background:**

Genome-wide comparisons of populations are widely used to explore the patterns of nucleotide diversity and sequence divergence to provide knowledge on how natural selection and genetic drift affect the genome. In this study we have compared whole-genome sequencing data from Atlantic and Pacific herring, two sister species that diverged about 2 million years ago, to explore the pattern of genetic differentiation between the two species.

**Results:**

The genome comparison of the two species revealed high genome-wide differentiation but with islands of remarkably low genetic differentiation, as measured by an *F*_*ST*_ analysis. However, the low *F*_*ST*_ observed in these islands is not caused by low interspecies sequence divergence (*d*_*xy*_) but rather by exceptionally high estimated intraspecies nucleotide diversity (*π*). These regions of low differentiation and elevated nucleotide diversity, termed high-diversity regions in this study, are not enriched for repeats but are highly enriched for immune-related genes. This enrichment includes genes from both the adaptive immune system, such as immunoglobulin, T-cell receptor and major histocompatibility complex genes, as well as a substantial number of genes with a role in the innate immune system, e.g. novel immune-type receptor, tripartite motif and tumor necrosis factor receptor genes. Analysis of long-read based assemblies from two Atlantic herring individuals revealed extensive copy number variation in these genomic regions, indicating that the elevated intraspecies nucleotide diversities were partially due to the cross-mapping of short reads.

**Conclusions:**

This study demonstrates that copy number variation is a characteristic feature of immune trait loci in herring. Another important implication is that these loci are blind spots in classical genome-wide screens for genetic differentiation using short-read data, not only in herring, likely also in other species harboring qualitatively similar variation at immune trait loci. These loci stood out in this study because of the relatively high genome-wide baseline for *F*_*ST*_ values between Atlantic and Pacific herring.

**Supplementary Information:**

The online version contains supplementary material available at 10.1186/s12864-024-10380-5.

## Background

Genetic screens based on whole-genome sequencing are widely used to identify loci underlying variation in phenotypic traits and ecological adaptation. For instance, this approach has been successfully used to identify hundreds of loci in the Atlantic herring (*Clupea harengus*) with striking genetic differentiation associated with adaptation to different ecological conditions and timing of reproduction [1]. Genetic screens comparing different species may also be used to identify loci that explain phenotypic differences or have contributed to speciation, if the species are sufficiently closely related and do not show extensive differentiation across the entire genome. This approach has been used to identify loci involved in adaptation in adaptive radiations of closely related species of cichlids [2, 3] and Darwin’s finches [4, 5], among many others. Here we present a genomic comparison of the Atlantic herring and its sister species, the Pacific herring (*Clupea pallasi*).

Atlantic and Pacific herring are both abundant species with key ecological roles in the North Atlantic and the North Pacific Oceans serving as links between primary plankton production and carnivorous fish, sea birds and sea mammals. The Atlantic herring is a schooling, long-distance migratory fish that is distributed along both the eastern and western shores of the North Atlantic Ocean. Its range extends southward to the English Channel and South Carolina [6, 7], and northeastwards to the White Sea and Barents Sea [8, 9]. In addition, populations of Atlantic herring are adapted to the brackish Baltic Sea. Atlantic herring primarily consists of winter–spring and summer–autumn spawning groups, and each population spawns once per year at a specific time [10, 11]. These populations return to their natal place for reproduction and spawn on rock bottoms [7]. The Pacific herring, in contrast, is distributed along both the eastern and western sides of the Pacific Ocean, ranging from the Chukchi Sea and the Beaufort Sea in the north to Baja California and the Sea of Japan in the south [12, 13]. Populations of Pacific herring also occur in the northeast European seas, including the White Sea, Pechora Sea, and Kara Sea [8, 9]. This species spawns once a year in late winter to spring [11]. Pacific herring also exhibits distinct spawning behaviors compared to Atlantic herring. It spawns in shallow, near-shore habitats on marine vegetation, in contrast with the deeper-spawning Atlantic herring [14]. The two species separated about two million years ago as estimated based on mitochondrial DNA sequence analysis [15]. However, there is a contact zone between the Northeast Atlantic and the European side of the subarctic basin where gene flow occurs [8]. Furthermore, a Pacific/Atlantic hybrid population is present in a subarctic fjord in Norway and has persisted for thousands of years [6].

Our previous whole-genome comparisons of Atlantic herring populations adapted to diverse ecological conditions—primarily regarding spawning season, water salinity at spawning place, and water temperature [10]—revealed striking genetic differentiation at hundreds of loci, with minimal differentiation observed at the remaining loci. This pattern deviates from what is expected under neutral evolution and is consistent with strong signatures of selection [1, 16].

Here, we performed a whole-genome scan for genetic differentiation between Atlantic and Pacific herring, to explore the pattern of genetic differentiation between the two species. However, identification of loci that have contributed to adaptive evolution subsequent to speciation was hampered by high genome-wide sequence divergence. In contrast, the screen turned out to be highly efficient to find loci with remarkably low interspecific genetic differentiation that tend to show high nucleotide diversity both within and between species. We show that these regions, referred to here as high-diversity regions, are highly enriched for genes involved in the immune system and that the remarkably low *F*_*ST*_ estimates are not due to low interspecies nucleotide divergence but rather to extremely high estimated intraspecies nucleotide diversity. We employ long-read sequencing to decipher genetic diversity at some of these regions.

## Results

### Negative correlation between interspecies differentiation and intraspecies nucleotide diversity

**Fig. 1:**
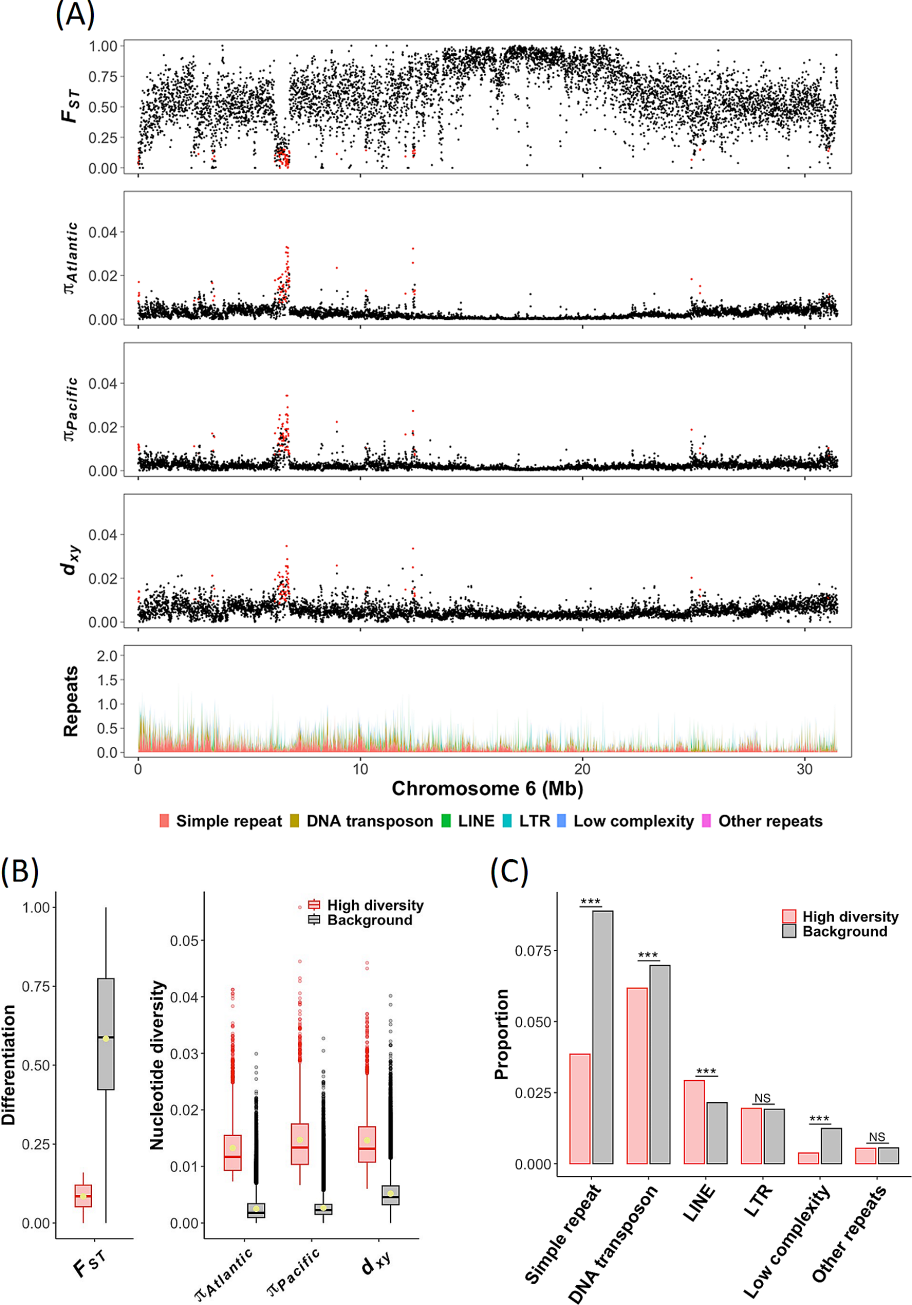
Distribution of population genetic parameters and repetitive elements calculated in nonoverlapping 5 kb windows in a comparison of Atlantic and Pacific herring. (**A**) Estimates of genetic differentiation (*F*_*ST*_), intraspecies nucleotide diversities in Atlantic (*π*_*Atlantic*_) and Pacific herring (*π*_*Pacific*_) and interspecies nucleotide diversity between Atlantic and Pacific herring (*d*_*xy*_) across chromosome 6 are displayed in the first to fourth tracks, respectively. Corresponding plots for all chromosomes are shown in Supplementary Fig. S1. Each dot represents a 5 kb window. Windows in the lower 5th percentile of the genome-wide distribution of *F*_*ST*_ and the top 5th percentile of the genome-wide distributions of *π*_*Atlantic*_ and *π*_*Pacific*_ are highlighted in red. The bottom track represents the cumulative proportion of repeats across the chromosome. (**B**) The box plots show the distributions of genetic differentiation (*F*_*ST*_) and nucleotide diversities (*π* and *d*_*xy*_) in the high-diversity regions (red) in comparison with the genomic background (black); the mean values are represented with yellow dots inside the boxes. All comparisons are statistically significant (*P* < 0.001). (**C**) The bar chart summarizes the proportion of coverage by different repeat superclasses within the high-diversity regions and background sequences. ***=*P* < 0.001; NS = not significant

We performed a genome-wide screening of genetic differentiation between Atlantic and Pacific herring, measured with *F*_*ST*_, in 5 kb windows using short-read data from individually sequenced samples (Supplementary Table S1). First, average pairwise genetic differentiation was generally high between Atlantic and Pacific herring (genome average *F*_*ST*_ = 0.58), most likely explained by the accumulation of nucleotide substitutions and genetic drift subsequent to speciation and limited gene flow between the two species. This pattern is illustrated for chromosome 6 in Fig. 1A (see Supplementary Fig. S1 for all chromosomes). Second, *F*_*ST*_ exhibits high variation across the chromosome, with most of the heterogeneity in the *F*_*ST*_ profile explained by variation in the recombination rate. *F*_*ST*_ values were negatively correlated with recombination rates (*r* = -0.51, *P* < 0.001; based on the Atlantic herring recombination map [17]), consistent with findings reported in previous studies [18–20]. Stretches of the chromosome with inflated *F*_*ST*_ values around the midpoint of the chromosome coincide with low recombining regions, and we hypothesize that these are centromeric regions. Third, the high *F*_*ST*_ baseline is interrupted by intervals of remarkably low *F*_*ST*_. For instance, a large interval with distinctly low *F*_*ST*_ around 6.5 Mb, along with several shorter intervals with the same pattern, is present on chromosome 6 (Fig. 1A). Additional examples of this pattern across all chromosomes are documented in Supplementary Fig. S1.

Nucleotide diversity within Atlantic (*π*_*Atlantic*_) and Pacific herring (*π*_*Pacific*_) and nucleotide divergence between the two species (*d*_*xy*_) were estimated within 5 kb windows based on short-read data (Fig. 1A and Supplementary Fig. S1). Genome-wide averages of *π* estimated for the two species were similar, with Pacific herring exhibiting a slightly higher level (*π*_*Pacific*_ = 0.0028) than Atlantic herring (*π*_*Atlantic*_ = 0.0026). The average *π* estimated in this study is comparable to that in previous reports (*π*_*Atlantic*_ = 0.0030; [1]). The genome-wide average for *d*_*xy*_ between Atlantic and Pacific herring was estimated at *d*_*xy*_ = 0.0053. The U-shaped pattern of the *π* and *d*_*xy*_ plots with low values in the middle of chromosome 6 most likely reflects variation in the recombination rate; the correlations of the recombination rate with *π*_*Atlantic*_, *π*_*Pacific*_ and *d*_*xy*_ were 0.45, 0.24 and 0.24, respectively (*P* < 0.001 for all comparisons). Further, we note that the regions with near-zero *F*_*ST*_ are not associated with reduced interspecies sequence divergence, the average *d*_*xy*_ for the lower 5th percentile of *F*_*ST*_ was 0.0064 in comparison with the genome-wide average of 0.0053. The low-*F*_*ST*_ regions are instead characterized by the very high denominator (intraspecies *π*) that drastically reduces *F*_*ST*_ estimates; the average *π*_*Atlantic*_ and *π*_*Pacific*_ for the lower 5th percentile of *F*_*ST*_ were 0.0058 and 0.0065, in comparison with the genome-wide averages of 0.0026 and 0.0028, respectively.

To further examine these signals and explore the underlying causes of the observed pattern, we extracted windows with *F*_*ST*_ value lower than the 5th percentile (*F*_*ST*_ < 0.16) and *π* values above the 95th percentile in each species (*π*_*Atlantic*_ > 0.0073 and *π*_*Pacific*_ > 0.0067). Based on these criteria, 2,030 windows (∼ 10 Mb in total) with particularly low *F*_*ST*_ and high nucleotide diversity in both species compared with the genomic background were identified (Fig. 1B). Throughout the paper, these regions are designated as high-diversity regions. *F*_*ST*_ has a moderate negative correlation with intraspecies nucleotide diversities estimated for the whole genome (-0.56 with *π*_*Atlantic*_ and − 0.42 with *π*_*Pacific*_; *P* < 0.001) but this relationship weakens in the high-diversity regions (-0.21 and − 0.24, respectively; *P* < 0.001). Estimates of nucleotide diversity within Atlantic and Pacific herring show a strong correlation (0.74; *P* < 0.001), as expected for closely related species. A strong positive correlation exists between *d*_*xy*_ and *π* in each species (0.77 for *π*_*Atlantic*_ and 0.70 for *π*_*Pacific*_; *P* < 0.001); this correlation is even stronger in the high-diversity regions (0.91 and 0.92, respectively; *P* < 0.001) (Supplementary Fig. S2).

### Repetitive elements are not enriched in high-diversity regions

Minimal interspecies differentiation in the high-diversity regions implies that a large proportion of the total variation occurs within species and thus, these signals can potentially be footprints of balancing selection. Balancing selection is characterized by maintaining genetic diversity above neutral expectations, thereby leading to low levels of genetic differentiation between populations in regions under selection [21, 22]. However, an alternative hypothesis is that the high-diversity regions are enriched for repetitive elements. Due to high copy number and sequence identity inherent in repetitive elements, these regions tend to yield unreliable SNP calls because of the difficulty to correctly align short reads to such regions. To assess whether the high-diversity regions were enriched for repeats, we calculated the proportion of each window occupied by different classes of repetitive elements. This analysis showed that the high-diversity regions were not enriched for repetitive elements (Fig. 1A). On the contrary, the average proportion of total repeats observed in the high-diversity regions (0.16) was lower than that in the genomic background (0.22; Welch’s t-test, *P* < 0.001). More specifically, simple repeats and low complexity repeats were less frequent across the high-diversity regions than in the rest of the genome (0.039 and 0.004 vs. 0.089 and 0.012, respectively; Welch’s t-test, *P* < 0.001) (Fig. 1C).

### High-diversity regions are highly enriched for immune-related genes

**Fig. 2:**
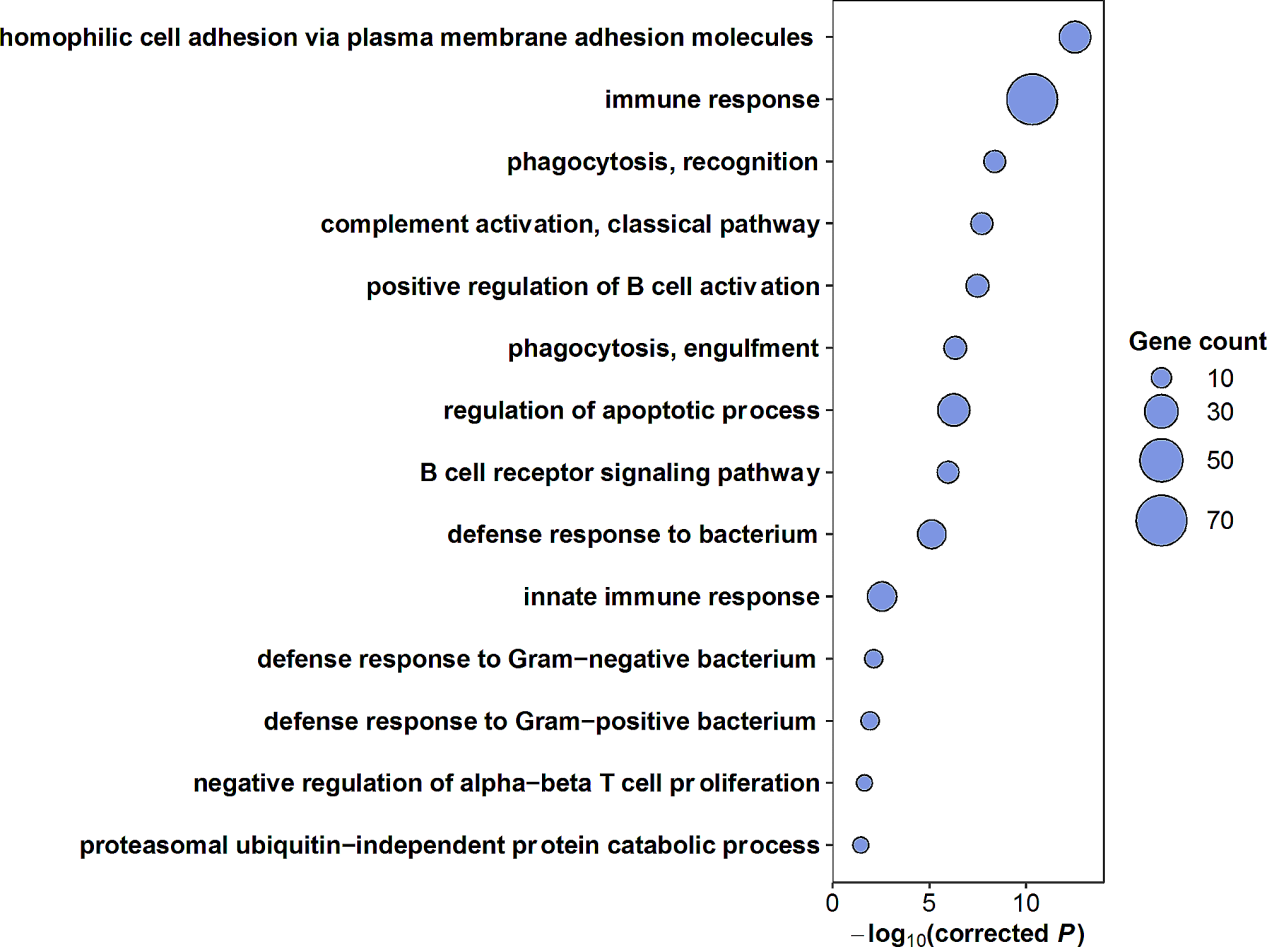
Significant biological process terms from GO overrepresentation analysis for genes located in high-diversity regions in Atlantic and Pacific herring. A Bonferroni-corrected significance threshold of *P* ≤ 0.01 was used. The size of each circle is proportional to the number of enriched genes in the corresponding GO category

We performed gene ontology (GO) term overrepresentation analysis for functional characterization of the genes located in the high-diversity regions. Out of 24,095 protein-coding genes in the Atlantic herring genome, 881 genes had full or partial overlap with the high-diversity regions. We noted that a much higher proportion of genes residing in the high-diversity regions lacked functional annotation compared with genes in the rest of the genome. Specifically, only 474 out of 881 (54%) protein-coding genes in the high-diversity regions had a GO annotation regardless of GO category, in contrast to 17,868 out of 24,095 genes (74%) in the entire genome (*χ*^2^ test, *P* < 0.001). To improve the functional annotation of the herring genes, we employed a pipeline developed by National Bioinformatics Infrastructure Sweden (NBIS) for functional annotation [23]. After the enhancement of the GO term annotations, a total of 21,345 genes were annotated genome-wide with at least one GO term irrespective of GO category, out of which 587 overlapped the high-diversity regions. For biological process (BP) term annotation, 17,783 genes were annotated genome-wide, including 270 genes within the high-diversity regions. Keeping only GO terms associated with a minimum of ten genes, 6,690 BP terms were assessed. The overrepresentation analysis was conducted with Weight01 algorithm implemented in the topGO R package [24], and revealed that the high-diversity regions were highly enriched for genes involved in immune-related processes (Fig. 2). This algorithm accounts for GO dependencies and hierarchical structure [25], but there are nevertheless some degrees of overlap between the top GO terms. These overlapping GO terms are closely related in the GO hierarchy. The interrelationships among the significant terms are illustrated in the GO graph in Supplementary Fig. S3A. The genes labelled with each term, and summary statistics of the GO terms, are compiled in Supplementary Table S2. The results for the overrepresented molecular function (MF) and cellular component (CC) terms are presented in Supplementary Table S2 and Supplementary Fig. S3 (B and C).

**Fig. 3:**
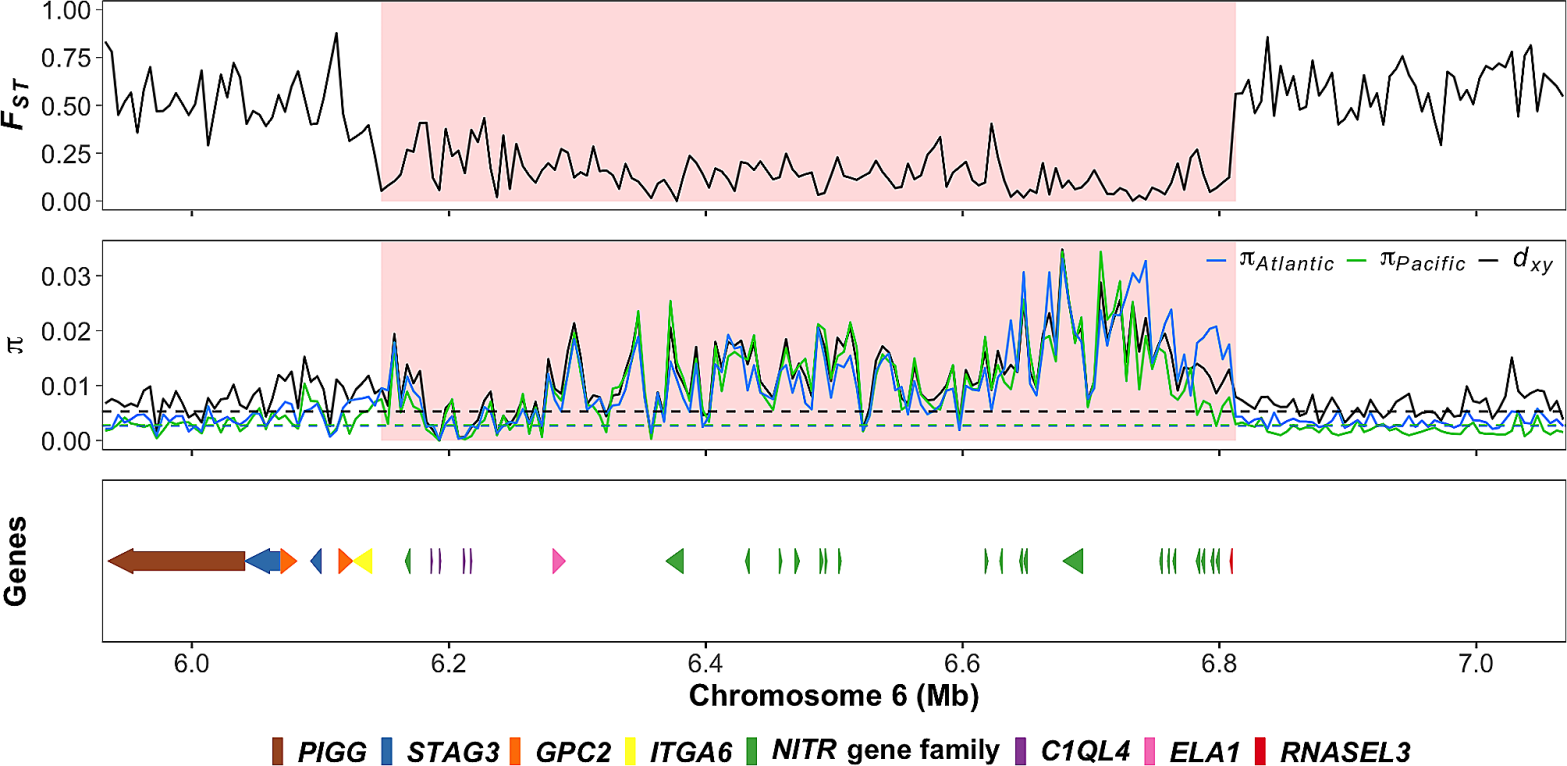
Zoomed-in plots of a high-diversity region on chromosome 6. The first track shows a marked *F*_*ST*_ drop in the highlighted region. The second track shows the elevated *π*_*Atlantic*_, *π*_*Pacific*_ and *d*_*xy*_ in the same region; dashed lines depict genome-wide averages. The shaded area in the plots depicts a high-diversity region. Gene organization on the reference assembly is presented in the bottom track (annotation source: NCBI *Clupea harengus* Annotation Release 102), indicating a cluster of *NITR* genes within the high-diversity region. Color code for genes is given below the figure

The above findings encouraged us to conduct a more detailed examination of some of the high-diversity regions to explore the structure and organization of the genes. For example, a 400 kb region around position 6.5 Mb on chromosome 6 comprises a cluster of genes coding for proteins that contain immunoglobulin-like domains (Fig. 3). These genes are homologous to novel immune-type receptor (*NITR*) genes in other teleosts [26, 27]. Additional examples of gene organization in high-diversity regions showing a similar pattern are presented in Supplementary Fig. S4, and a comprehensive list of high-diversity regions can be found in Supplementary Table S3. Based on these analyses, we conclude that the most characteristic feature of the high-diversity regions in the comparison of Atlantic and Pacific herring is that they are composed of clusters of immune-related genes.

To assess the prevalence of the observed pattern in natural populations of other species, we looked for signals of low differentiation and high nucleotide diversity in previously published data from fish. We identified comparable signals among the radiations of Midas cichlid [2] and in stickleback [28] that contain clusters of immune trait genes similar to those observed in herring (Supplementary Table S4), suggesting that despite being a repeated pattern in teleost fish, such regions have been largely overlooked, or at least left undescribed, in previous comparative genomic studies.

### PacBio long-read data reveal extensive copy number variation in high-diversity regions

**Fig. 4:**
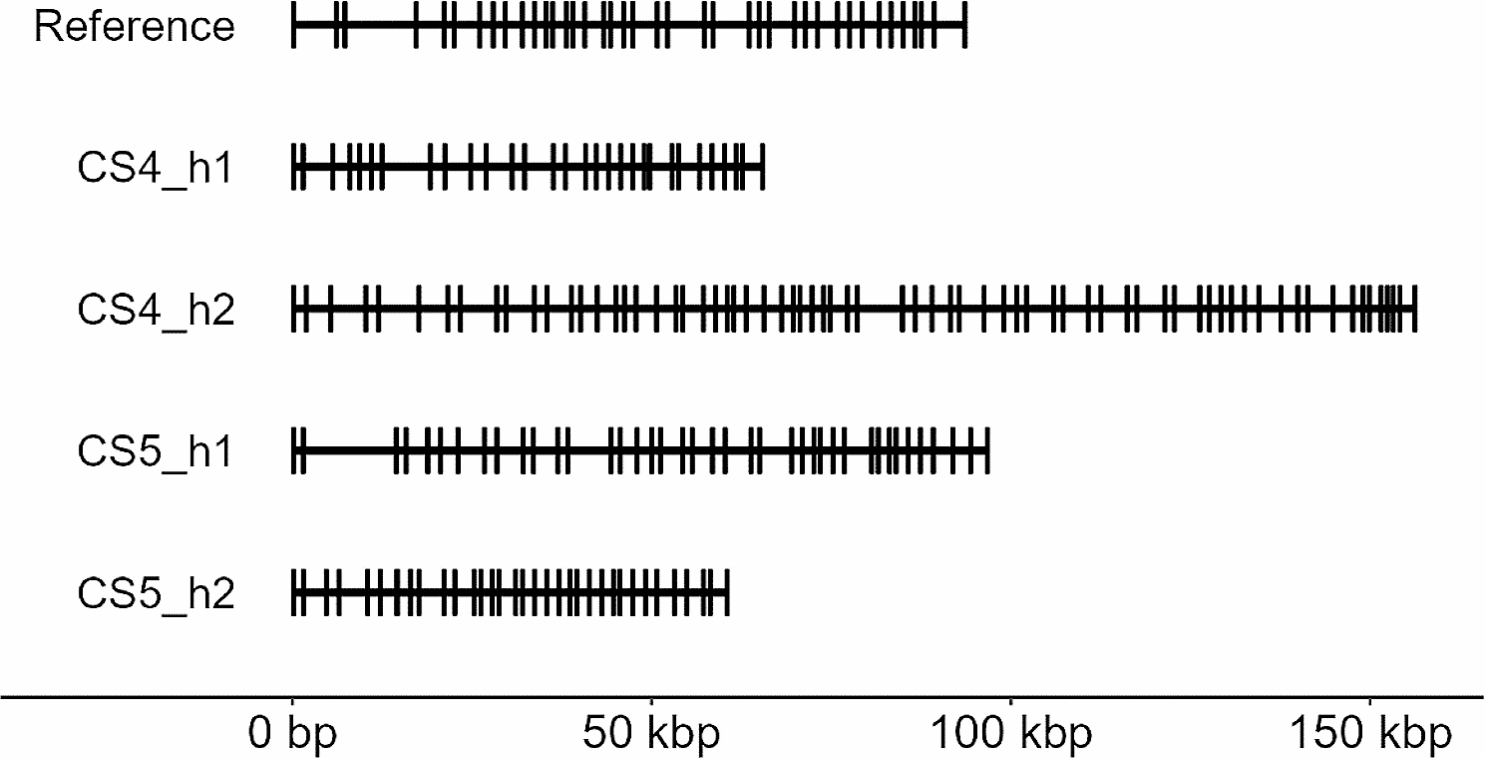
Genome organization of the *IGHV* region on the Atlantic herring reference assembly (Chr1:1,882,931-1,976,669) and on four PacBio-based haplotype assemblies. Each vertical black line represents a gene, thus illustrating the copy number variation at the *IGHV* locus

The analysis of genetic diversity at clusters of closely related genes using short-read data is challenging, partially because the genome assembly may be incorrect with collapsed copy number variation even if the assembly is based on long read data as is the case for the Atlantic herring reference assembly [17]. In addition, errors may occur because of the difficulty to align short reads to the correct copy – in fact, due to structural variation, there is often no “correct copy” for a subset of reads. We therefore generated *de novo* genome assemblies of two Atlantic herring individuals based on PacBio HiFi long reads to overcome this problem (genome statistics in Table 1). We generated nearly 22 Gb of sequencing data with an average read length of 20 kb for each individual. This allowed us to study copy number variation as well as to more accurately estimate levels of nucleotide diversity. Here we present a detailed analysis of three representative high-diversity regions containing clusters of (i) immunoglobulin heavy variable (*IGHV*), (ii) CD300e molecule (*CLM2*), and (iii) interferon-induced protein with tetratricopeptide repeats 10 (*IFIT10*). These regions are representative of the complex nature of genomic regions harboring immune genes and include multigene/single gene family as well as innate/adaptive immune genes. We also considered the contiguity of assembly contigs and gene annotation when selecting these regions. We first annotated the genes and noted the presence of multiple gene copies along with pseudogenes composed of incomplete or disrupted coding sequences (Table 2). The *IGHV* locus varied between 30 and 70 gene copies distributed across a region ranging in estimated size from 64 kb to 160 kb on different haplotypes (Fig. 4A).

**Table 1:** Statistics for haplotype-phased genome assemblies of two Atlantic herring individuals (CS4 and CS5) based on PacBio HiFi sequencing

Assemblies^a^	Genome size (Mb)	BUSCO^b^ (%)	No. contigs	N50 (kb)
CS4_h1	776.3	92.8	3,253	693.1
CS4_h2	751.3	92.8	2,539	731.1
CS5_h1	768.1	91.6	3,817	545.0
CS5_h2	745.9	91.6	3,037	569.8

^a^h1 and h2 correspond to haplotypes 1 and 2 from the same individual

^b^Benchmarking Universal Single-Copy Orthologs

**Table 2:** Gene copy numbers of *IGHV*, *CLM2*, and *IFIT10* in three high-diversity regions in five assemblies^a^

Assemblies	IGHV		CLM2		IFIT10	
	No. of genes (ψ^c^)	Size (kb)	No. of genes (ψ^c^)	Size (kb)	No. of genes (ψ^c^)	Size (kb)
Reference^b^	40 (0)	94	4 (1)	29	2 (0)	22
CS4_h1	30 (0)	70	4 (3)	47	2 (1)	42
CS4_h2	70 (0)	160	4 (1)	53	2 (1)	49
CS5_h1	40 (0)	100	4 (4)	74	2 (2)	58
CS5_h2	34 (0)	64	4 (4)	66	2 (1)	29

^a^The total number of gene copies in each region includes both functional and null copies, with the latter representing alleles carrying inactivating mutations. The number of pseudogenes (ψ) is given in parentheses

^b^Atlantic herring reference assembly [17]

^c^ψ = Number of pseudogenes

**Fig. 5:**
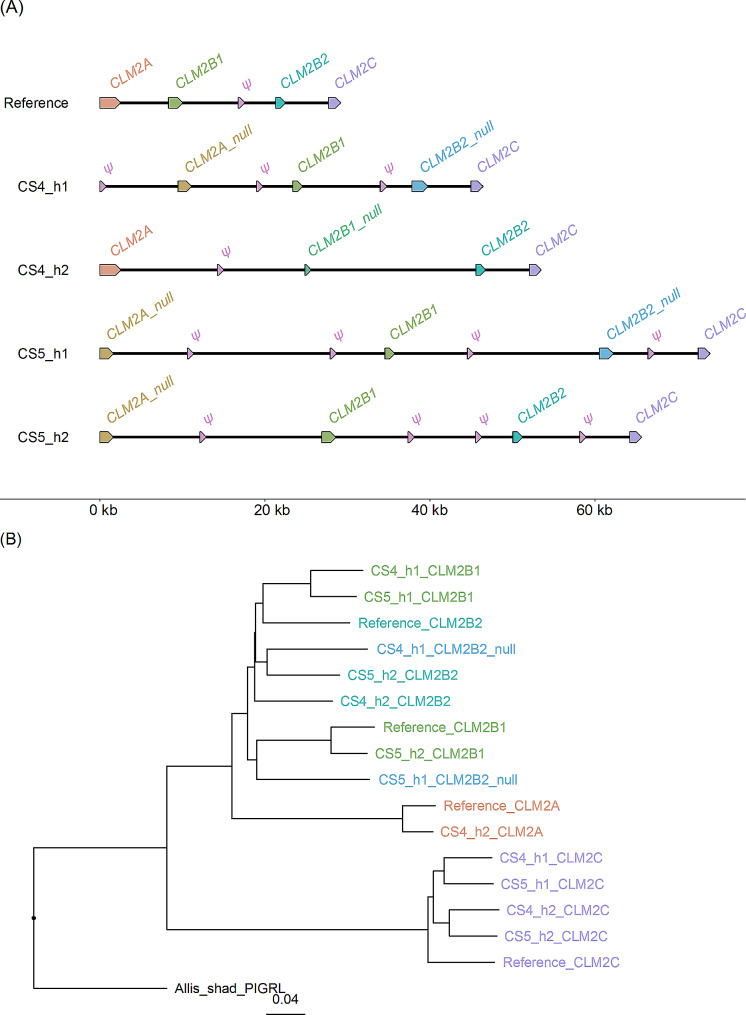
Copy number variation of the cluster of *CLM2* genes on chromosome 22 in the region 18,433,425–18,434,439 bp of the reference assembly. (**A**) Genomic organization of *CLM2* genes on the Atlantic herring reference assembly and four PacBio-based haplotype assemblies. *CLM2A_null* lacks exon1 and intron1 which is present in *CLM2A*. Similarly, *CLM2B1_null* in CS4_h2 lacks exon1 and intron1 which is present in *CLM2B1*. *CLM2B2_null* in CS4_h1 and CS5_h1 has one nonsense mutation in exon2. Sequences referred to as pseudogenes (*ψ*) contain only fragments of *CLM2* coding sequences. (**B**) Phylogenetic tree of *CLM2* coding nucleotide sequences. The color code is the same as in Fig. 5A

**Fig. 6:**
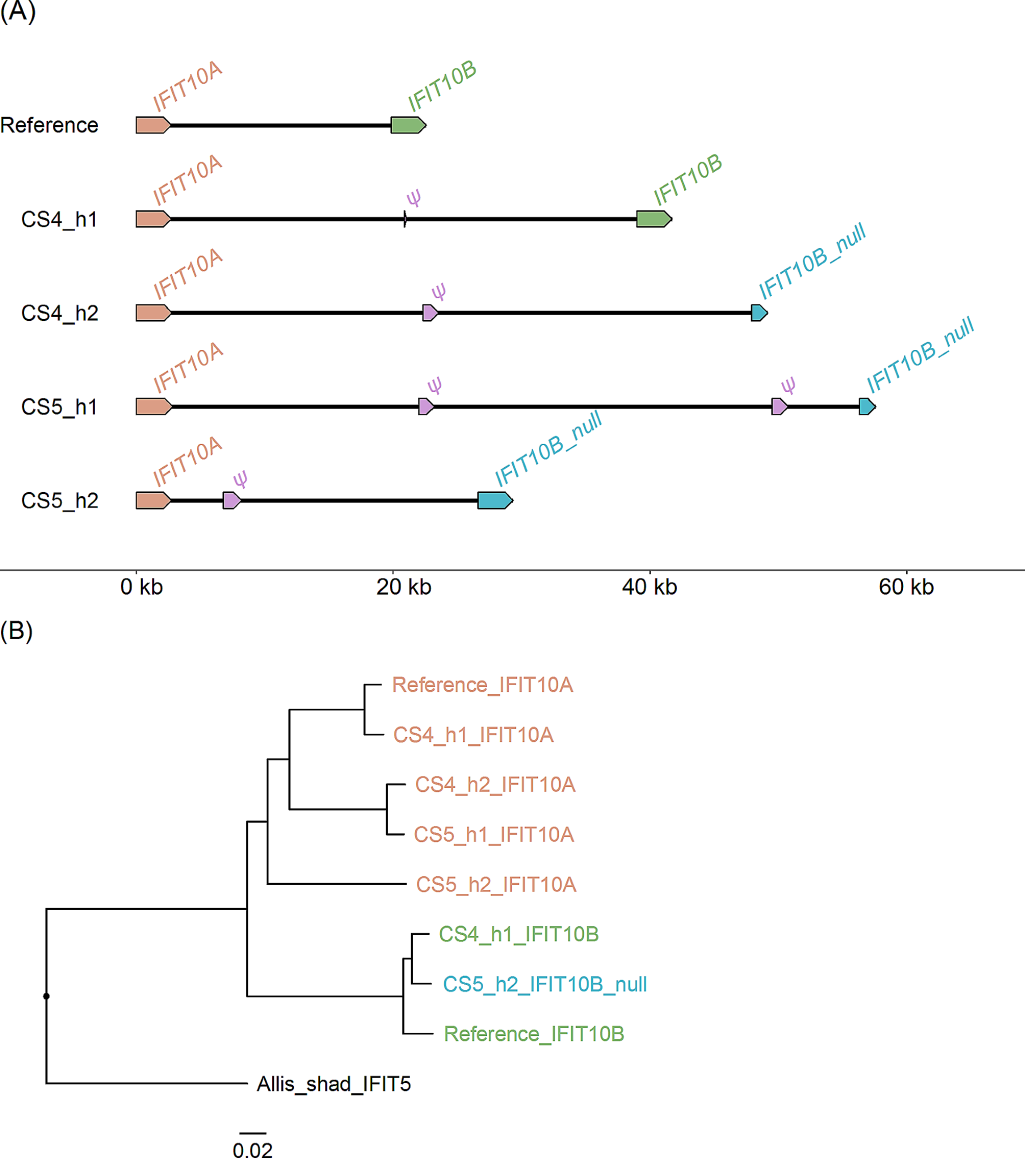
Copy number variation of the cluster of *IFIT10* genes on chromosome 23 in the region 12,114,407 − 12,116,709 bp of the reference assembly. (**A**) Genomic organization of *IFIT10* genes on five haplotypes. *IFIT10B*_*null* in CS4_h2 and CS5_h1 had multiple stop codons throughout the gene length. *IFIT10B_null* has a deletion of four bp. (**B**) Phylogenetic tree of *IFIT10* coding nucleotide sequences with Allis shad *IFIT5* as an outgroup. The color code is the same as in Fig. 6A

The Atlantic herring reference assembly [17] contains a cluster of four *CLM2* genes and one *CLM2* pseudogene on chromosome 22. The PacBio assemblies of this region also contained four full length copies and the number of *CLM2* pseudogenes varied from one to four (Fig. 5). Furthermore, the lengths of the PacBio assemblies were all longer than the reference assembly suggesting that duplicated sequences may have been collapsed in the reference assembly. A phylogenetic tree analysis revealed that the *CLM2C* sequences formed a distinct group whereas the genes designated *CLM2A*, *CLM2B1*, and *CLM2B2* were all closely related and did not form three distinct allelic series. Thus, it is not possible to align short read sequences to the correct copy (based on genomic location) of *CLM2A/B* genes. The assembled regions containing a pair of *IFIT10* genes on chromosome 23 were all longer on the new PacBio assemblies compared with the reference assembly, and the former contained an *IFIT10* pseudogene lacking in the reference assembly (Fig. 6).

We estimated nucleotide diversity (*π*) for the coding sequences of *CLM2* and *IFIT10* using short-read and long-read data. For this purpose, we used genomic context to ensure that allelic sequences were compared. This revealed a notable difference in the *π* values estimated from long-read data compared to those estimated based on short-read data. However, several of these genes had still much higher nucleotide diversity than the genome average of 0.003 (Table 3).

**Table 3:** Nucleotide diversity (*π*) based on coding sequences (CDS) of *CLM2* and *IFIT10* genes

Genes	CDS length (bp)	π (long reads)^c^	π (short reads)^d^
*CLM2B1*	738^a^	0.054 (3)	0.015
*CLM2B2*	690	0.031 (4)	0.027
*CLM2C*	696	0.014 (4)	0.012
*IFIT10A*	1440	0.031 (4)	0.064
*IFIT10B*	1374^b^	0.001 (2)	0.046

^a^One sequence is 741 bp and three are 738 bp

^b^*IFIT10B* in CS5_h2 is 1370 bp and has a 4 bp deletion compared with other sequences

^c^Nucleotide diversity calculated using sequences extracted from PacBio assemblies from CS4 and CS5 samples. The number of sequences used in the *π* calculation is given in parentheses and indicates the copies with complete coding sequences, excluding truncated copies

^d^Nucleotide diversity calculated using the same samples based on short-read data with pixy [29] and the AllSites VCF file

## Discussion

In the present study, a window-based scan of sequence divergence between Atlantic and Pacific herring revealed an unexpected set of low-*F*_*ST*_, high-*d*_*xy*_ regions that clearly stood out against the genomic background, which is characterized by high *F*_*ST*_ and moderate *d*_*xy*_. Close inspection of these high-diversity regions unveiled a striking enrichment of immune-related genes, typically forming multigene family clusters. To our knowledge, no previous study has highlighted such regions, but our re-examination of publicly available data revealed a similar enrichment of immune trait genes in regions with low *F*_*ST*_ between populations and high nucleotide diversity in Midas cichlids [2] and sticklebacks [28]. These findings imply that the observed pattern is widespread, yet has remained largely unexplored as regards to its contribution to genome evolution during speciation and adaptation.

We show that clusters of genes involved in adaptive immunity, such as immunoglobulin, T-cell receptor, and major histocompatibility genes, as well as innate immunity, such as novel immune-type receptor, tripartite motif, tumor necrosis factor receptor, and NOD-like receptor genes are highly enriched at the high-diversity regions detected in this study (Supplementary Table S3). Immune response genes are among the fastest evolving genes and species/lineage-specific clades of different categories of immune genes are commonly observed [30]. This is a reasonable explanation as to why a relatively large proportion of the genes in the high-diversity regions lack functional annotation. Furthermore, the presence of several pseudogenes in the multigene clusters is consistent with these loci following a gene birth-and-death model [31, 32]. Many of the genes present in the high-diversity regions belong to the innate immune system, an observation consistent with the fact that in fish, defense mechanisms against pathogens are skewed toward innate immunity [33, 34], while in mammals, adaptive immune responses are more important for immune protection [35, 36]. Previous studies have also noted lineage/species-specific gene family expansions of genes involved in innate immunity in teleosts relative to other vertebrates, and related these expansions to the evolutionary success of the teleost lineage; this includes genes encoding complement factors [37], toll-like receptors (*TLR*) [38, 39], novel immune-type receptor (*NITR*) [26, 40], NACHT-domain and leucine-rich-repeat-containing (*NLR*) [41, 42], tripartite motif proteins (*TRIM*) [41, 43], CC chemokines [44] and interferons [45]. Our observations demonstrate that immune-related gene families also underwent rapid and dynamic evolution in the two sister species of herring studied here.

Studying genomic regions harboring multigene family clusters proves to be challenging with the use of short sequence reads because of the difficulty to distinguish allelic and non-allelic sequences with high sequence identities. Thus, population genetic parameters, such as *F*_*ST*_, estimated based on short-read sequencing data are prone to be biased at such regions, and hence should be interpreted with caution. We therefore used PacBio long-read data to make it possible to separate haplotypes of homologous chromosomes and identified fine-scale pattern of these complex regions. This analysis revealed copy number variation (CNV), and other re-arrangements, at many of the high-diversity regions. In fact, none of the four haplotypes deduced from two individuals had an identical structure to another haplotype in the three gene regions studied in detail (Figs. 4, 5 and 6). The analysis showed that the extensive genetic diversity at immune-related genes in the Atlantic herring is due to a combination of copy number variation and nucleotide diversity between alleles.

Previous studies have detected a large number of loci contributing to ecological adaptation in Atlantic herring [1, 15]. These studies were facilitated by the extremely low genetic differentiation at neutral loci providing an unusually high signal-to-noise ratio in genetic screens. However, the present study has revealed blind spots in these genetic screens, namely the high-diversity regions described here, that have likely played a critical role in the evolutionary history of these two species. The high nucleotide diversity within populations at these loci will result in low *F*_*ST*_ estimates also when comparing different populations of Atlantic herring adapted to different environmental conditions, such as those in the marine Atlantic Ocean compared with the brackish Baltic Sea. It is clear that long-read sequencing is required to fully explore the genetic diversity at these loci and how it may contribute to genetic adaptation in herring, and many other vertebrate species.

The Atlantic herring is one of the world’s most abundant vertebrates and a single school may be composed of a billion individuals [46], making them an attractive target for pathogens. The Pacific herring is also an abundant species and its spawning in shallow water may favour spreading of pathogens. It is likely that the genetic diversity at immune-related genes described in this study contributes to the genetic defense against pathogens and thus to overall fitness.

## Conclusions

This study has revealed copy number variation and high nucleotide diversity at genes related to both adaptive and innate immune system in Atlantic herring. This is probably a widespread pattern among teleosts. We document that population genetic parameters estimated using short-read sequencing data are unreliable for these regions due to their complexity. They also appear as blind spots in genome scans for regions of genetic differentiation based on *F*_*ST*_ statistics due to the very high within population nucleotide diversity. We demonstrated that the long-read sequencing approach has the capacity to allow accurate estimation of genetic diversity in these regions and provide new insights into their contribution to ecological adaptation in herring, and in other teleosts.

## Materials and methods

### Short-read data alignment and variant calling

The Atlantic and Pacific herring samples used in the population genetic analysis were collected in our previous studies [1, 15, 16]. The samples of Atlantic herring were collected from both the Northeast and Northwest Atlantic Ocean, and the Pacific herring samples were captured close to Vancouver. Information about the location and date of sampling, water salinity and spawning season is provided in Supplementary Table S1. Genomic DNA was isolated by standard methods, and sequencing libraries were constructed for each individual. Whole-genome sequencing was carried out with Illumina short-read sequencing and paired-end reads were generated. More detailed procedures for whole-genome sequencing are described in the references provided in Table S1.

We mapped short-reads for each individual to the Atlantic herring reference assembly Ch_v2.0.2 [17] with the BWA-MEM algorithm [47]. Variant calling was performed using DNAseq pipeline in Sentieon suite [48]. We used the Haplotyper algorithm to call variants for each sample, and subsequently used the GVCFtyper algorithm to perform the joint variant calling of all samples. In the variant calling process, all sites, encompassing both variants and invariants, were retained in the output VCF file, denoted as the AllSites VCF. Following the variant calling, indels were excluded, and sites with the following filtering criteria were removed: FS > 60.0, MQRankSum < -12.5, MQ < 40.0, QD < 2.0, ReadPosRankSum < -8.0, and SOR > 3.0. Additionally, genotypes with GQ < 20, DP < 2, and DP > 100 were filtered out. This filtering was performed using the VariantFiltration and SelectVariants tools in GATK [49].

### Estimation of population genetic parameters

Population genetic statistics including population differentiation and within and between nucleotide diversity were estimated within nonoverlapping 5 kb windows along each chromosome using pixy [29]. Fixation index (*F*_*ST*_) was estimated using Weir and Cockerham’s approach [50]. Average per-site nucleotide differences between all pairs of sequences were calculated within populations (nucleotide diversity, *π*) and between populations (nucleotide divergence, *d*_*xy*_) within 5 kb windows. Pixy avoids underestimation of nucleotide diversity by using AllSites VCF file. Unbiased estimation of nucleotide diversity is achieved by accounting for missing data in the calculation. Separate files for *F*_*ST*_, *π* and *d*_*xy*_ and for different chromosomes were combined and windows with a missing value for any of the parameters were excluded from the final file. Windows with *F*_*ST*_ values below the 5th percentile and *π* values above the 95th percentile in each of the two species were extracted for downstream analyses.

### Genome screening for repetitive elements

We screened the entire Atlantic herring reference genome [17] for repetitive elements with RepeatMasker [51]. The resulting repetitive elements were classified into superclasses and summarized over the same 5 kb windows that were used for computing diversity parameters. For each window, we calculated the proportion containing different superclasses of repeats.

### Gene ontology term overrepresentation analysis

To perform overrepresentation analysis for Gene ontology (GO) terms, we included all protein-coding genes that fully or partially overlapped the windows with *F*_*ST*_ values falling below the 5th percentile and *π* values exceeding the 95th percentile in each species. To improve the functional annotation of genes in the herring genome, we used a Nextflow-based pipeline for functional annotation developed by the National Bioinformatics Infrastructure Sweden (NBIS) [23]. This pipeline starts with performing BLAST [52] searches for protein sequences extracted from GFF coordinates against protein database (e-value cutoff was set to 1e-6) and requires UniProt protein fasta file as reference to find the best BLAST matches. Only manually curated proteins (SwissProt proteins) from vertebrates were used. With this approach, it assigns a name to the gene and a description (corresponding to the gene product) to the transcripts. In the next step, it runs InterProScan software package to functionally characterize the genes and assign them functional annotations, including GO terms annotation. The NBIS FunctionalAnnotation pipeline in particular played a role in the improvement of the number of characterized genes with assigned names which increased from 16,499 to 22,579. To further enhance the Gene Ontology (GO) annotation, we obtained orthologs of Atlantic herring genes (with orthology confidence = 1) from BioMart, and assigned the GO annotations of these orthologs, sourced from eleven fish species (zebrafish, stickleback, Atlantic salmon, rainbow trout, Japanese medaka, Asian bonytongue, electric eel, goldfish, Nile tilapia, orange clownfish, common carp) and human, to the corresponding Atlantic herring genes. We then built the final gene-to-GO map file which links each gene identifier with one or more GO terms. This file was used to test the overrepresentation of GO terms.

We used the topGO R package [24] for GO term overrepresentation analysis, which provides the possibility to use a custom Gene-to-GO map. The default algorithm, weight01, was used. This algorithm takes the GO topology into account and tests the significance of each GO term depending on its related terms [25]. The GO hierarchical structure was read into TopGO from GO.db package. Since the analysis was based on gene count, and no gene score was available, Fisher’s exact test was implemented to evaluate the overrepresentation of GO terms. GO terms with less than 10 annotated genes were excluded from the analysis. The number of terms and genes incorporated in the analysis are summarized in Table 4.

**Table 4:** Numbers of genes and nodes incorporated into the GO term overrepresentation analysis

GO category	No. background genes^a^	No. genes in high-diversity regions^b^	No. nodes^c^
Biological process (BP)	17,783	270	6,690
Molecular function (MF)	19,837	503	1,319
Cellular component (CC)	18,024	323	832

^a^GO annotated genes from the genome subjected to overrepresentation analysis

^b^GO annotated genes from the high-diversity regions subjected to overrepresentation analysis

^c^GO terms with at least ten annotated genes

All distribution plots for population genetic parameters and the proportion of repeats were generated using the ggplot2 R package [53]. Plots of gene organization in the high-diversity regions on the reference assembly were created using the gggenes R package [54], and GO graphs were visualized with the topGO R package [24].

### Generation of PacBio long-read data

We generated PacBio HiFi sequencing data from two individuals of Atlantic herring, CS4 and CS5, captured on November 25, 2019 in the Celtic Sea. Testis samples were collected and flash-frozen on-site. DNA was extracted using a Circulomics Nanobind Tissue Big DNA Kit (NB-900-701-001) and sequenced using Pacific Biosciences (PacBio) High Fidelity (HiFi) technology. HiFi reads are known to have high accuracy (above 99.8%) and long contiguity (average read length of 13.5 kb) [55] hence we used them to build haploid *de novo* genome assemblies using hifiasm (v0.16.1-r375) [56]. The four haplotypes from the PacBio assemblies from the two individuals along with the sequences from the reference assembly were used for further analysis.

We selected three regions from the genome scan to characterize the high-diversity regions. As the GO analysis showed a strong enrichment of immune response genes in the high-diversity regions, we selected three representative regions that illustrate the complex nature of immune genes including multigene/single gene family and innate/adaptive immune genes. We also considered the contiguity of assembly contigs and gene annotation for selecting these regions. One of the regions encoded for immunoglobulin heavy variable (*IGHV*) multigene family on chromosome 1, and the other two regions were annotated as containing single genes and without an indication of a multigene family on chromosomes 22 and 23, with Ensembl IDs as ENSCHAG00000003891 and ENSCHAG00000015470, respectively. The latter two genes lacked gene name in the Ensembl database, hence to name them, we used nucleotide BLAST [52] to find the most similar gene sequence. CMRF35-like molecule 2 (*CLM2*) and interferon-induced protein with tetratricopeptides 10 (*IFIT10*) were found to be highly similar to ENSCHAG00000003891 and to ENSCHAG00000015470, respectively.

To check for additional copies of these genes, we used nucleotide BLAST [52] and MUMmer [57] aligner using the coding sequences. It resulted in total 40 *IGHV*, four *CLM2*, and two *IFIT10* genes, and a few pseudogenes. Additional *IGHV* genes lacked Ensembl IDs, hence these were manually annotated. Additional *CLM2* genes were ENSCHAG00000003799, ENSCHAG00000003851, and ENSCHAG00000003927, while additional *IFIT10* was ENSCHAG00000015478. We named the copies of these additional genes in the order they occurred on the genome – *CLM2A, CLM2B1, CLM2B2*, and *CLM2C* on Chr22, and *IFIT10A* and *IFIT10B* on Chr23.

To annotate these genes on the PacBio assemblies, we used LiftOff [58] with additional “-polish and -copies” parameters. Because *IGHV* is a multigene family, LiftOff algorithm was not successful in annotating all *IGHV* genes, hence we performed manual curation and used nucleotide BLAST [52] to find homologous sequences using manually annotated reference sequences as queries.

We used gggenomes R package [59] to visualize genomic organization of the annotated regions. The phylogenetic tree was constructed using ape R package [60] and visualized using ggtree [61]. Homologous genes from the clupeid species *Alosa alosa* (allis shad) were used as an outgroup to root the tree. Genes that were null due to incomplete sequence lengths were excluded for the construction of phylogenetic tree.

To assess the reliability of *π* estimates for the high-diversity regions derived from short-read data, we calculated *π* for the coding sequences of *CLM2* and *IFIT10* genes, once based on short-read data and once using long-read data. To calculate *π* based on short-read data, we utilized pixy [29] with the AllSites VCF file, keeping only CS4 and CS5 samples in the calculation. For accurate estimation of *π* from long-read data, coding sequences only from untruncated gene copies were extracted from four haploid PacBio assemblies obtained from CS4 and CS5. The sequences were subsequently aligned using the msa R package [62], and *π* was calculated using the pegas R package [63].

### Electronic supplementary material

Below is the link to the electronic supplementary material.

Supplementary Material 1: Additional file 1: Table S1.

Supplementary Material 2: Additional file 2: Fig. S1.

Supplementary Material 3: Additional file 3: Fig. S2.

Supplementary Material 4: Additional file 4: Fig. S3.

Supplementary Material 5: Additional file 5: Table S2.

Supplementary Material 6: Additional file 6: Fig. S4.

Supplementary Material 7: Additional file 7: Table S3.

Supplementary Material 8: Additional file 8: Table S4.

## Data Availability

The raw long-read sequencing data used in this study are deposited in the NCBI Sequence Read Archive (SRA) under the BioProject accession number PRJNA1023520.
