# Supplementary material for: Copy number variation and elevated genetic diversity at immune trait loci in Atlantic and Pacific herring
Source: BMC Genomics. 2024 May 10;25:459. doi: 10.1186/s12864-024-10380-5 (PMC11088111; doi:10.1186/s12864-024-10380-5)
Supplement: Supplementary file 1 — Supplementary Material 1: Additional file 1: Table S1. [file 12864_2024_10380_MOESM1_ESM.pdf]

Table S1. Samples of Atlantic and Pacific herring used in short-read data analysis

Identifier	Location	Latitude	Longitude	Region[a]	Spawning season	Sample size	Salinity (ppt)	Date	Reference
i17	Bergen	N60°35′	E05°00′	Northeast Atlantic Ocean	Spring	8	33	20130522	Martinez Barrio et al. 2016
i34	Celtic Sea	N51° 59'	W6° 51'	Northeast Atlantic Ocean	Winter	3	35	20151201	Fan et al. 2020
i37	Downs	N51° 34'	E1° 90'	Northeast Atlantic Ocean	Winter	3	35	20161212	Fan et al. 2020
i10	Isle of Man	N54° 6'	W4° 37'	Northeast Atlantic Ocean	Autumn	3	35	20150930	Fan et al. 2020
i7	North Sea	N58°06′	E6°10′	Northeast Atlantic Ocean	Autumn	3	35	19790805	Lamichhaney et al. 2017
i31	Norway	N67° 46'	E9°47'	Northeast Atlantic Ocean	Spring	3	35	20170220	Fan et al. 2020
i1	Canada(Bonavista Bay)	N48°49′	W53°20′	Northwest Atlantic Ocean	Autumn	2	35	20140625	Lamichhaney et al. 2017
i26	Canada(Fortune Bay)	N47°17′	W55°38′	Northwest Atlantic Ocean	Spring	2	35	20140526	Lamichhaney et al. 2017
i5	Canada(German Banks)	N43°16′	W66°18′	Northwest Atlantic Ocean	Autumn	2	35	20140828	Lamichhaney et al. 2017
i30	Canada(Inner Baie Des Chaleurs)	N48°00′	W65°51′	Northwest Atlantic Ocean	Spring	2	35	20140508	Lamichhaney et al. 2017
i3	Canada(Northumberland Strait)	N45°44′	W62°36′	Northwest Atlantic Ocean	Autumn	2	35	20140916	Lamichhaney et al. 2017
i28	Canada(Northumberland Strait)	N46°19′	W64°09′	Northwest Atlantic Ocean	Spring	2	35	20140506	Lamichhaney et al. 2017
i58	Vancouver, Strait of Georgia	N49°28′	W123°15′	Pacific Ocean	Spring	6	35	20121124	Lamichhaney et al. 2017

[a]The samples from the Atlantic Ocean represent Atlantic herring while the samples from the Pacific Ocean represent Pacific herring
