# Supplementary material for: Copy number variation and elevated genetic diversity at immune trait loci in Atlantic and Pacific herring
Source: BMC Genomics. 2024 May 10;25:459. doi: 10.1186/s12864-024-10380-5 (PMC11088111; doi:10.1186/s12864-024-10380-5)
Supplement: Supplementary file 2 — Supplementary Material 2: Additional file 2: Fig. S1. [file 12864_2024_10380_MOESM2_ESM.pdf]

Figure S1. Genome-wide distribution of population genetic parameters and repetitive elements in a comparison between Atlantic and Pacific herring for chromosome 1-26. Distribution across chromosome 6 is presented in Figure 1A in the main text. The parameters were estimated within nonoverlapping 5 kb windows. Track one to four display population differentiation (F_{ST}), intra-population nucleotide diversities in Atlantic ($\pi_{Atlantic}$) and Pacific herring ($\pi_{Pacific}$) and inter-population nucleotide diversity between Atlantic and Pacific herring (d_{xy}), respectively. Each dot represents a 5 kb window. Red dots represent windows in the lower 5th percentile of F_{ST} and the top 5th percentile of $\pi_{Atlantic}$ and $\pi_{Pacific}$. The cumulative proportion of repeats is displayed in the bottom track, and the color code of repeats superfamilies is given below it.

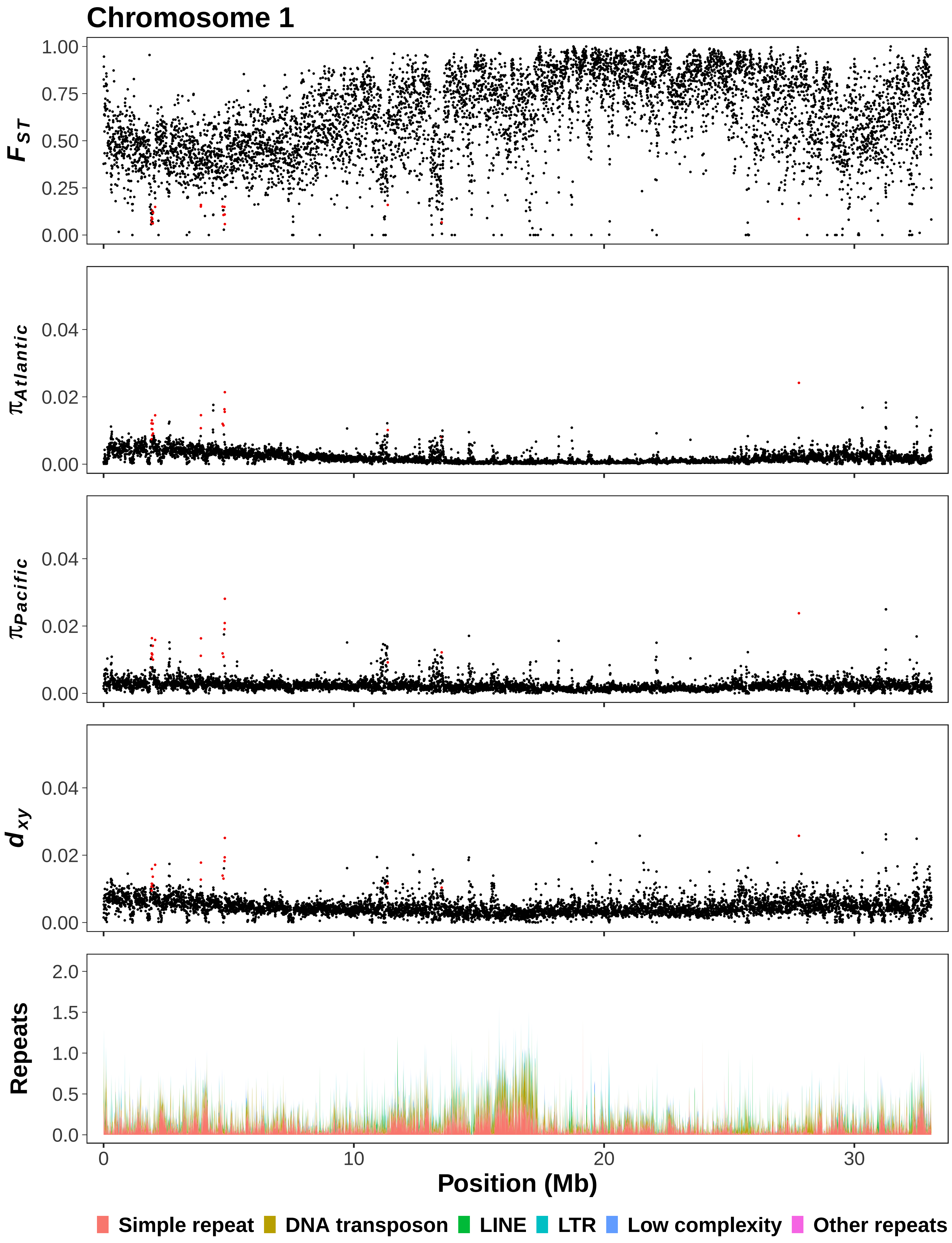

Chromosome 1
F_{ST}
1.00
0.75
0.50
0.25
0.00
$\pi_{Atlantic}$
$\pi_{Pacific}$
d_{xy}
0.04
0.02
0.00
Repeats
2.0
1.5
1.0
0.5
0.0
0
10
20
30
Position (Mb)
Simple repeat
DNA transposon
LINE
LTR
Low complexity
Other repeats

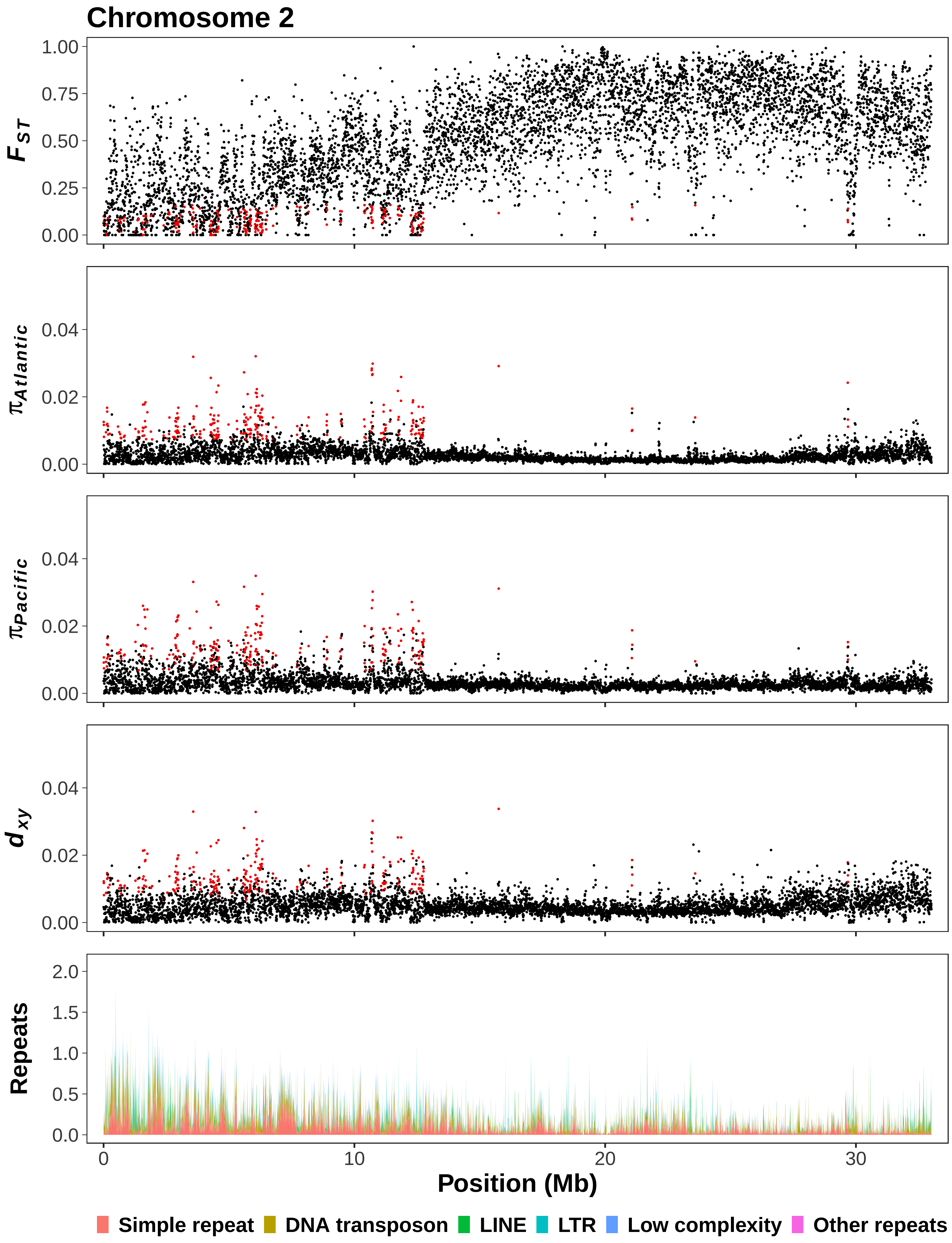

Chromosome 2
F_{ST}
1.00
0.75
0.50
0.25
0.00
$\pi_{Atlantic}$
0.04
0.02
0.00
$\pi_{Pacific}$
0.04
0.02
0.00
d_{xy}
0.04
0.02
0.00
Repeats
2.0
1.5
1.0
0.5
0.0
0
10
20
30
Position (Mb)
Simple repeat
DNA transposon
LINE
LTR
Low complexity
Other repeats

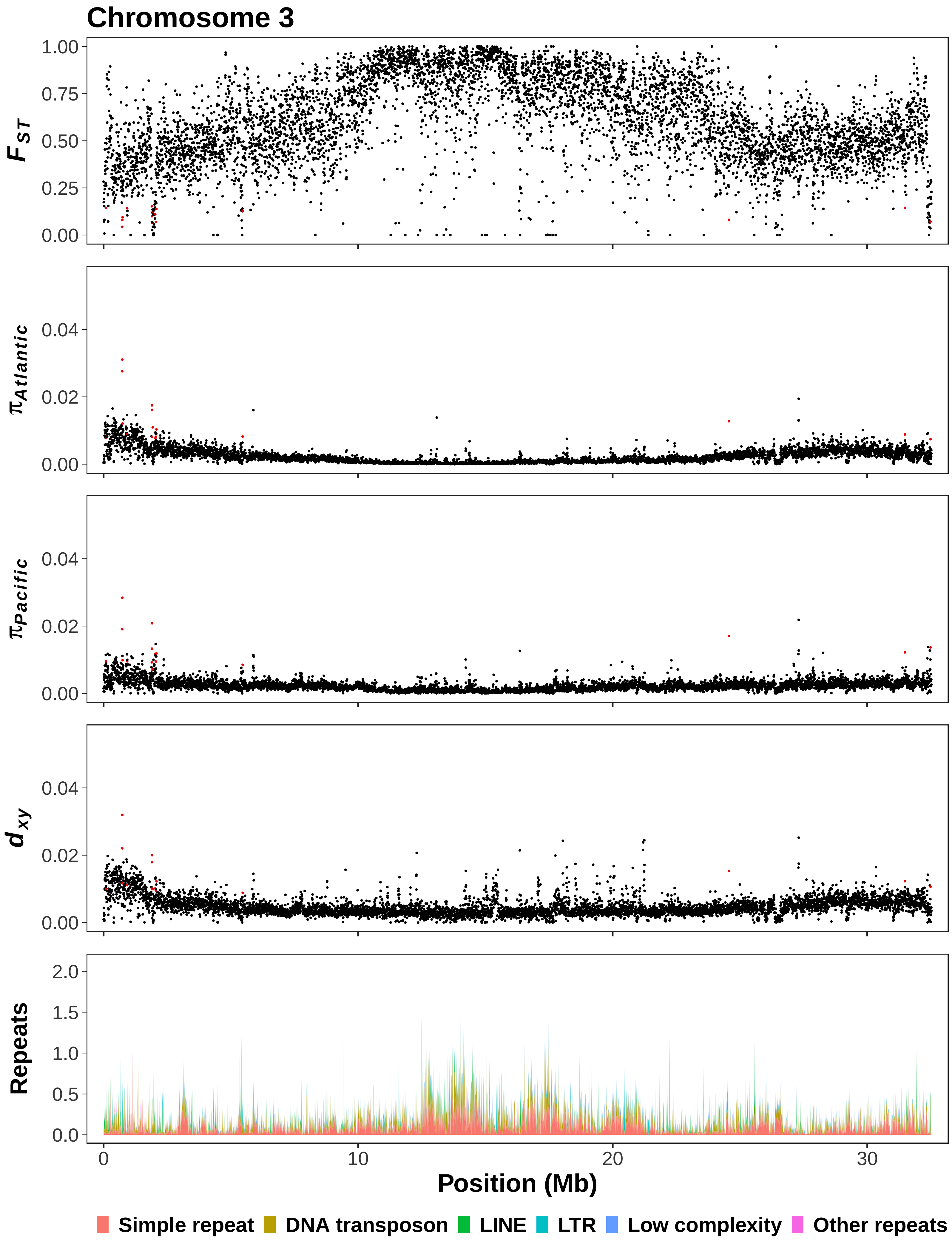

Chromosome 3
F_{ST}
1.00
0.75
0.50
0.25
0.00
$\pi_{Atlantic}$
$\pi_{Pacific}$
d_{xy}
0.04
0.02
0.00
Repeats
2.0
1.5
1.0
0.5
0.0
0
10
20
30
Position (Mb)
Simple repeat
DNA transposon
LINE
LTR
Low complexity
Other repeats

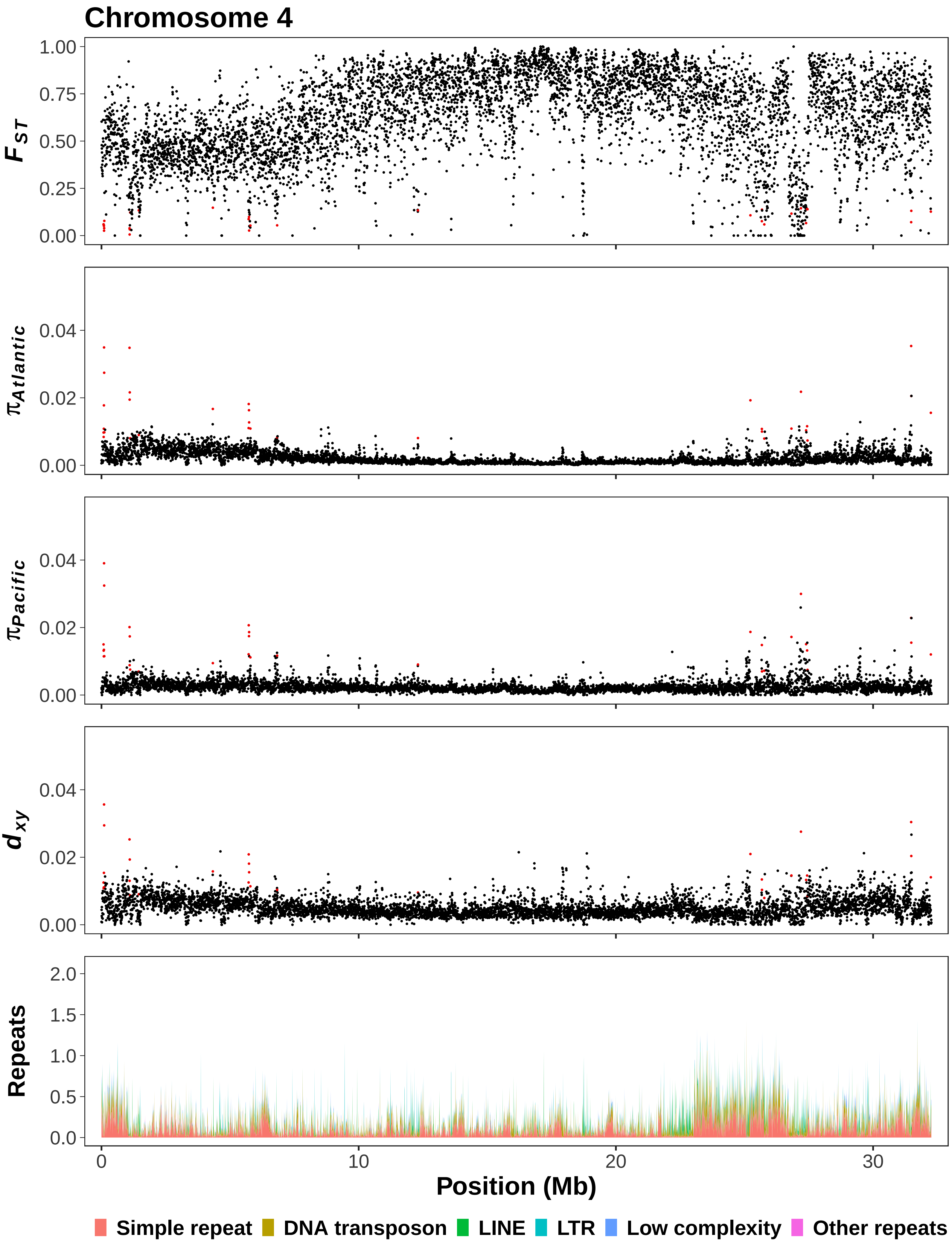

Chromosome 4
F_{ST}
1.00
0.75
0.50
0.25
0.00
$\pi_{Atlantic}$
0.04
0.02
0.00
$\pi_{Pacific}$
0.04
0.02
0.00
d_{xy}
0.04
0.02
0.00
Repeats
2.0
1.5
1.0
0.5
0.0
0
10
20
30
Position (Mb)
Simple repeat
DNA transposon
LINE
LTR
Low complexity
Other repeats

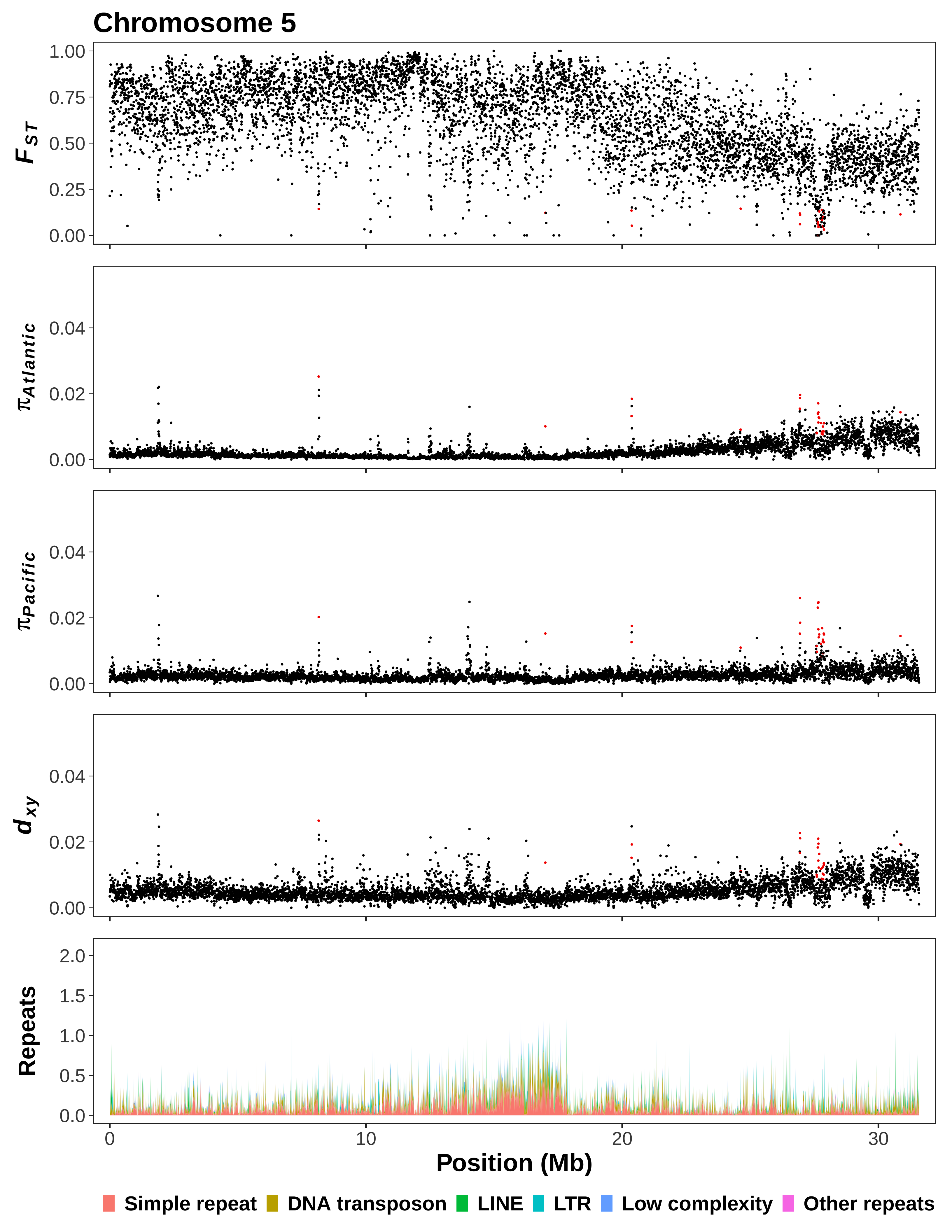
Chromosome 5
F_{ST}
1.00
0.75
0.50
0.25
0.00
$\pi_{Atlantic}$
0.04
0.02
0.00
$\pi_{Pacific}$
0.04
0.02
0.00
d_{xy}
0.04
0.02
0.00
Repeats
2.0
1.5
1.0
0.5
0.0
0
10
20
30
Position (Mb)
Simple repeat
DNA transposon
LINE
LTR
Low complexity
Other repeats

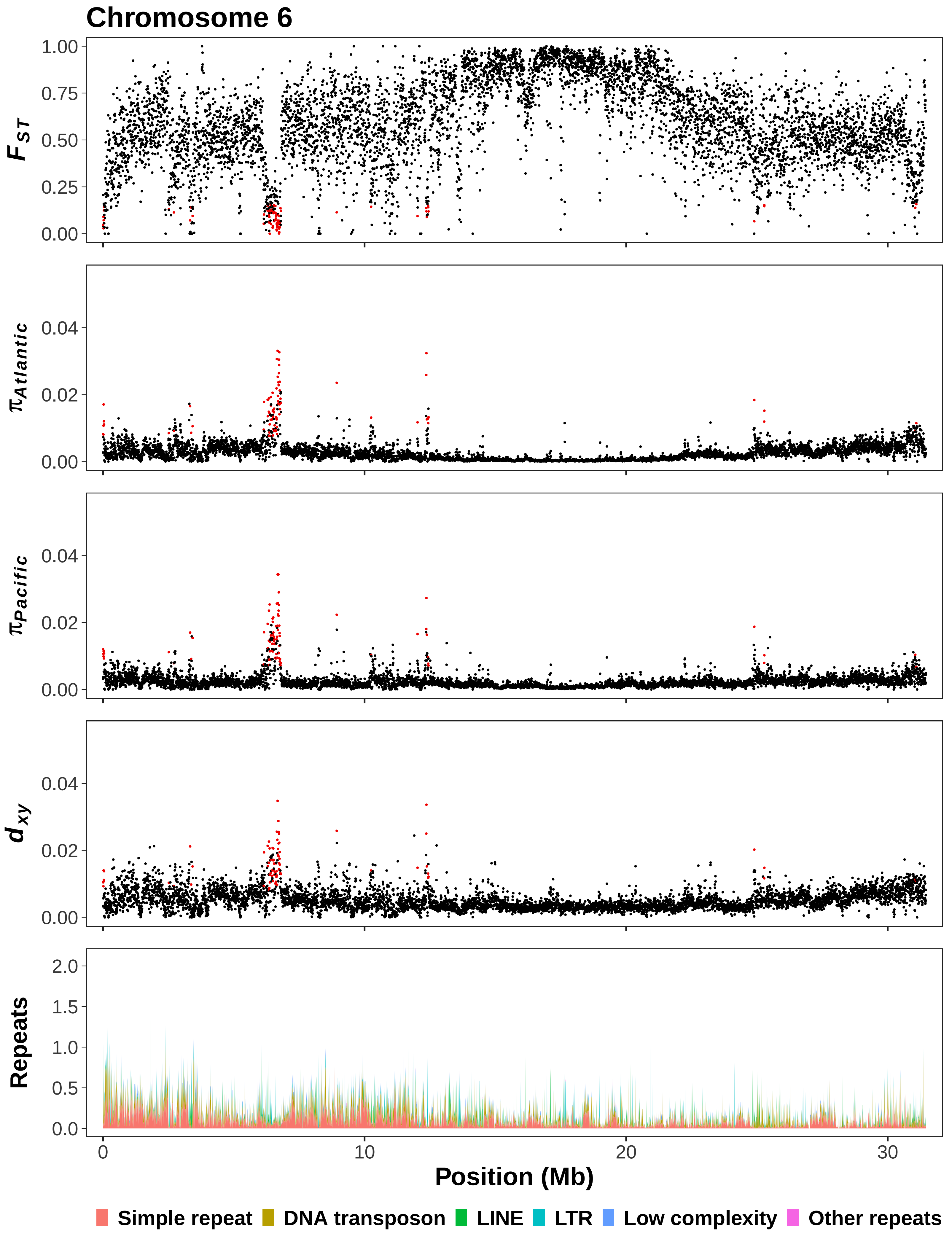

Chromosome 6
F_{ST}
1.00
0.75
0.50
0.25
0.00
$\pi_{Atlantic}$
0.04
0.02
0.00
$\pi_{Pacific}$
0.04
0.02
0.00
d_{xy}
0.04
0.02
0.00
Repeats
2.0
1.5
1.0
0.5
0.0
0
10
20
30
Position (Mb)
Simple repeat
DNA transposon
LINE
LTR
Low complexity
Other repeats

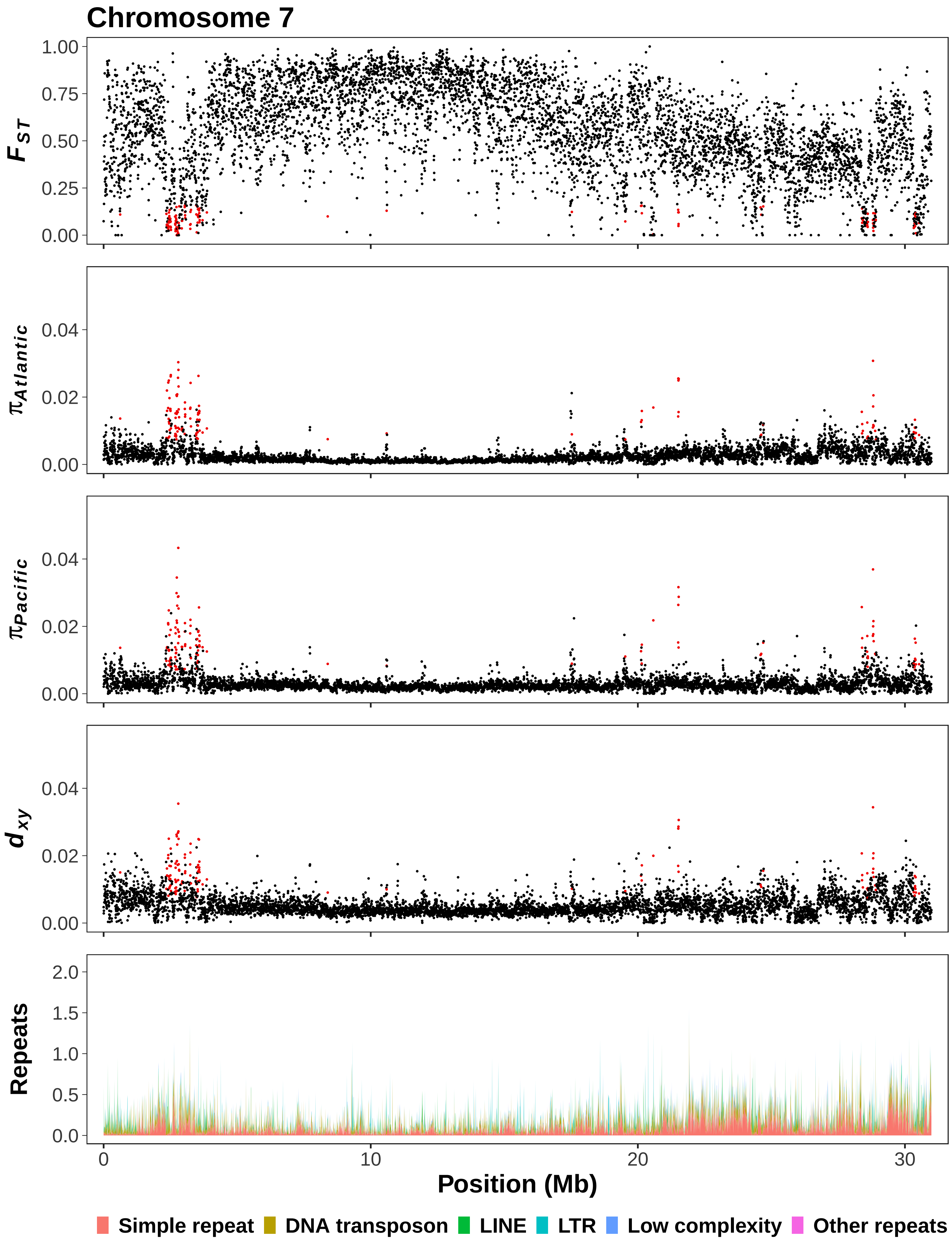

Chromosome 7
F_{ST}
1.00
0.75
0.50
0.25
0.00
$\pi_{Atlantic}$
0.04
0.02
0.00
$\pi_{Pacific}$
0.04
0.02
0.00
d_{xy}
0.04
0.02
0.00
Repeats
2.0
1.5
1.0
0.5
0.0
0
10
20
30
Position (Mb)
Simple repeat
DNA transposon
LINE
LTR
Low complexity
Other repeats

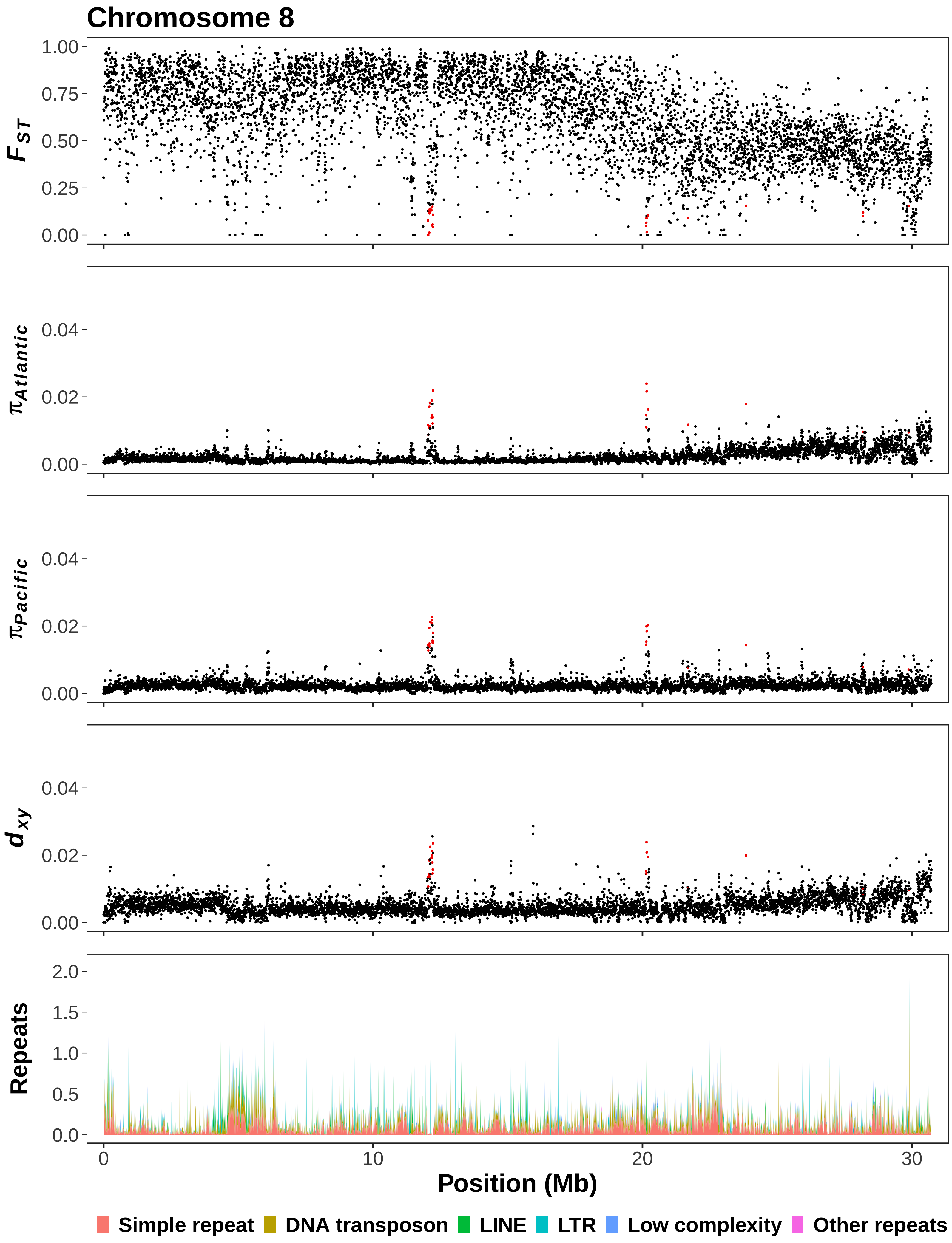

Chromosome 8
F_{ST}
1.00
0.75
0.50
0.25
0.00
$\pi_{Atlantic}$
0.04
0.02
0.00
$\pi_{Pacific}$
0.04
0.02
0.00
d_{xy}
0.04
0.02
0.00
Repeats
2.0
1.5
1.0
0.5
0.0
0
10
20
30
Position (Mb)
Simple repeat
DNA transposon
LINE
LTR
Low complexity
Other repeats

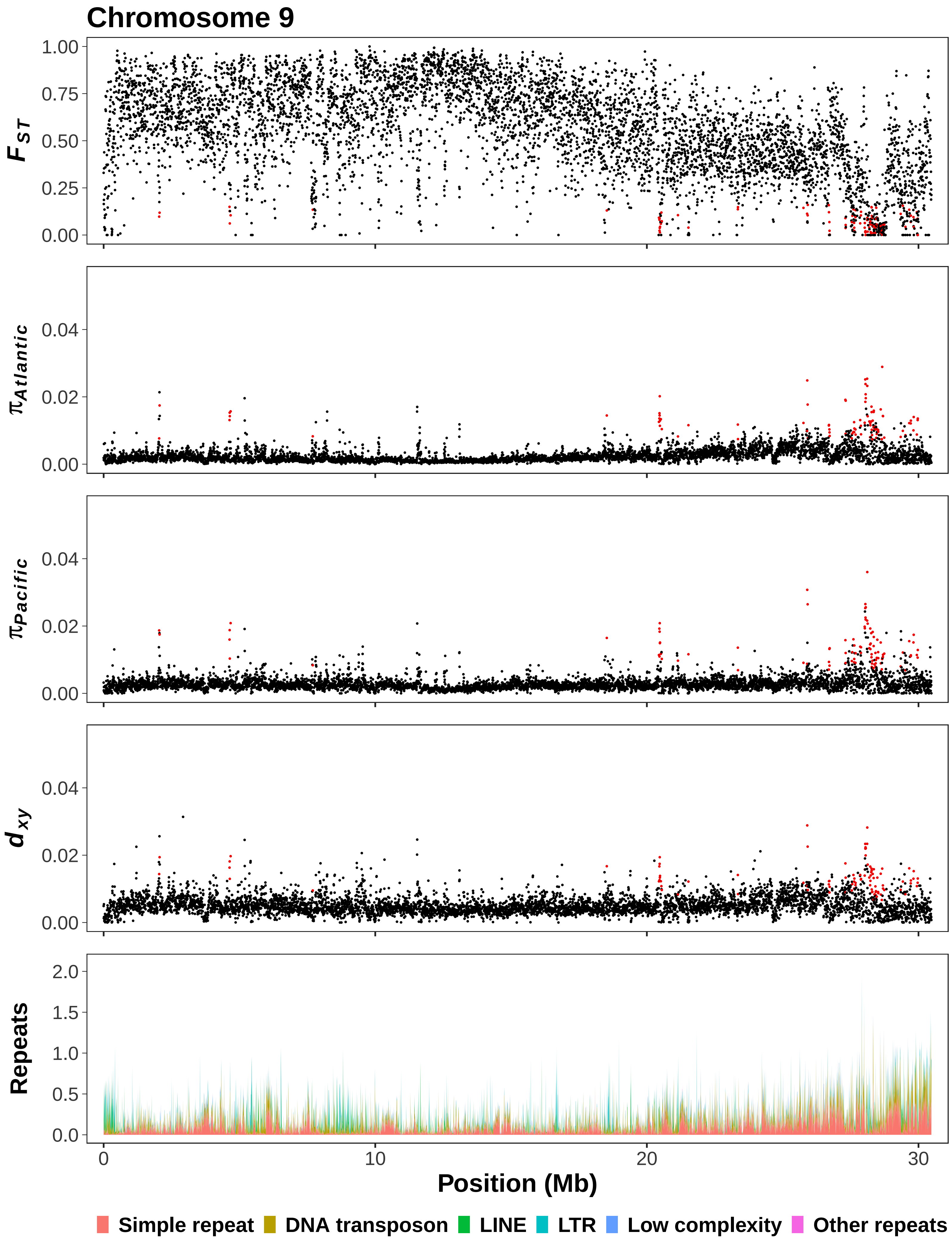

Chromosome 9
F_{ST}
$\pi_{Atlantic}$
$\pi_{Pacific}$
d_{xy}
Repeats
Position (Mb)
Simple repeat
DNA transposon
LINE
LTR
Low complexity
Other repeats

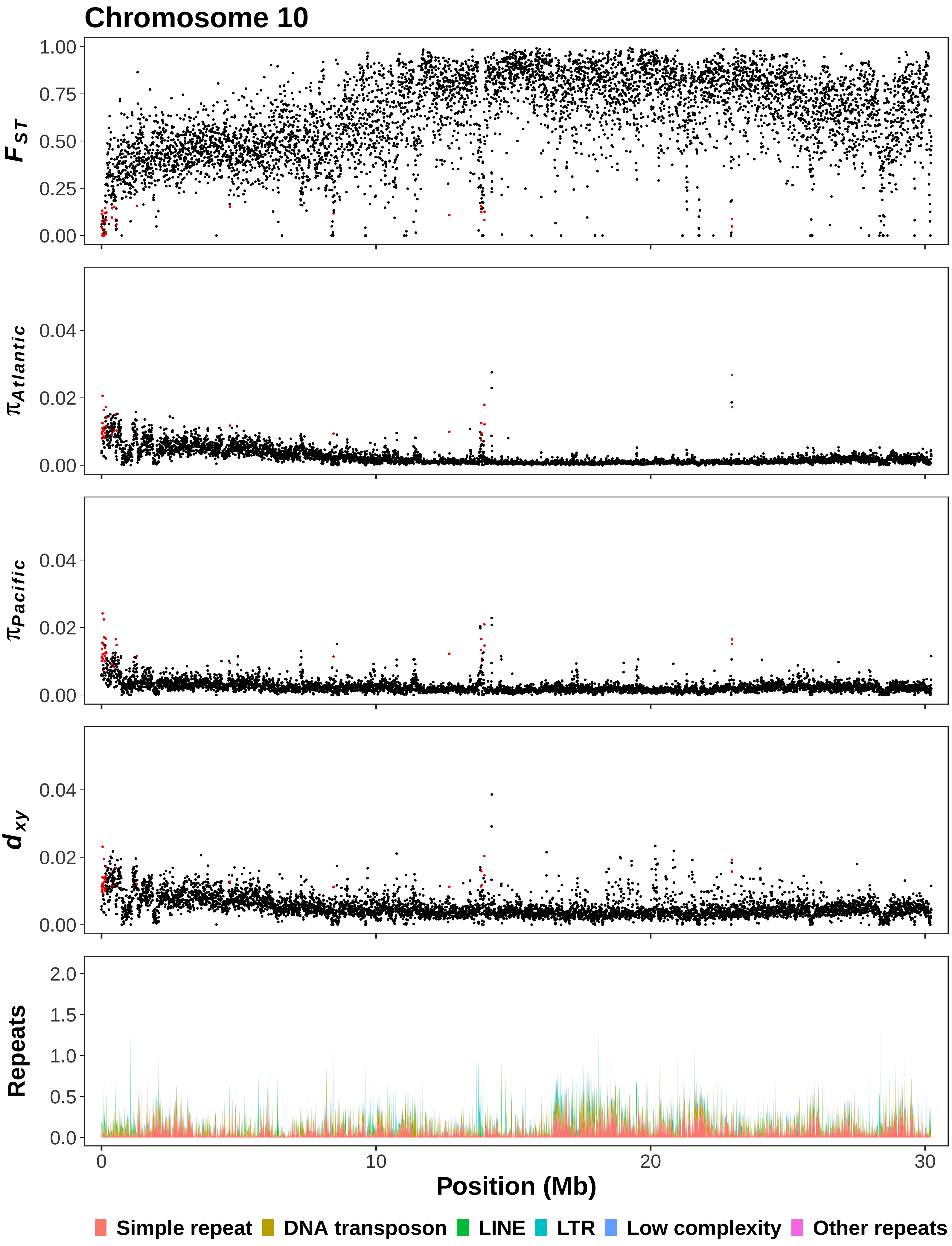

Chromosome 10
F_{ST}
1.00
0.75
0.50
0.25
0.00
$\pi_{Atlantic}$
0.04
0.02
0.00
$\pi_{Pacific}$
0.04
0.02
0.00
d_{xy}
0.04
0.02
0.00
Repeats
2.0
1.5
1.0
0.5
0.0
0
10
20
30
Position (Mb)
Simple repeat
DNA transposon
LINE
LTR
Low complexity
Other repeats

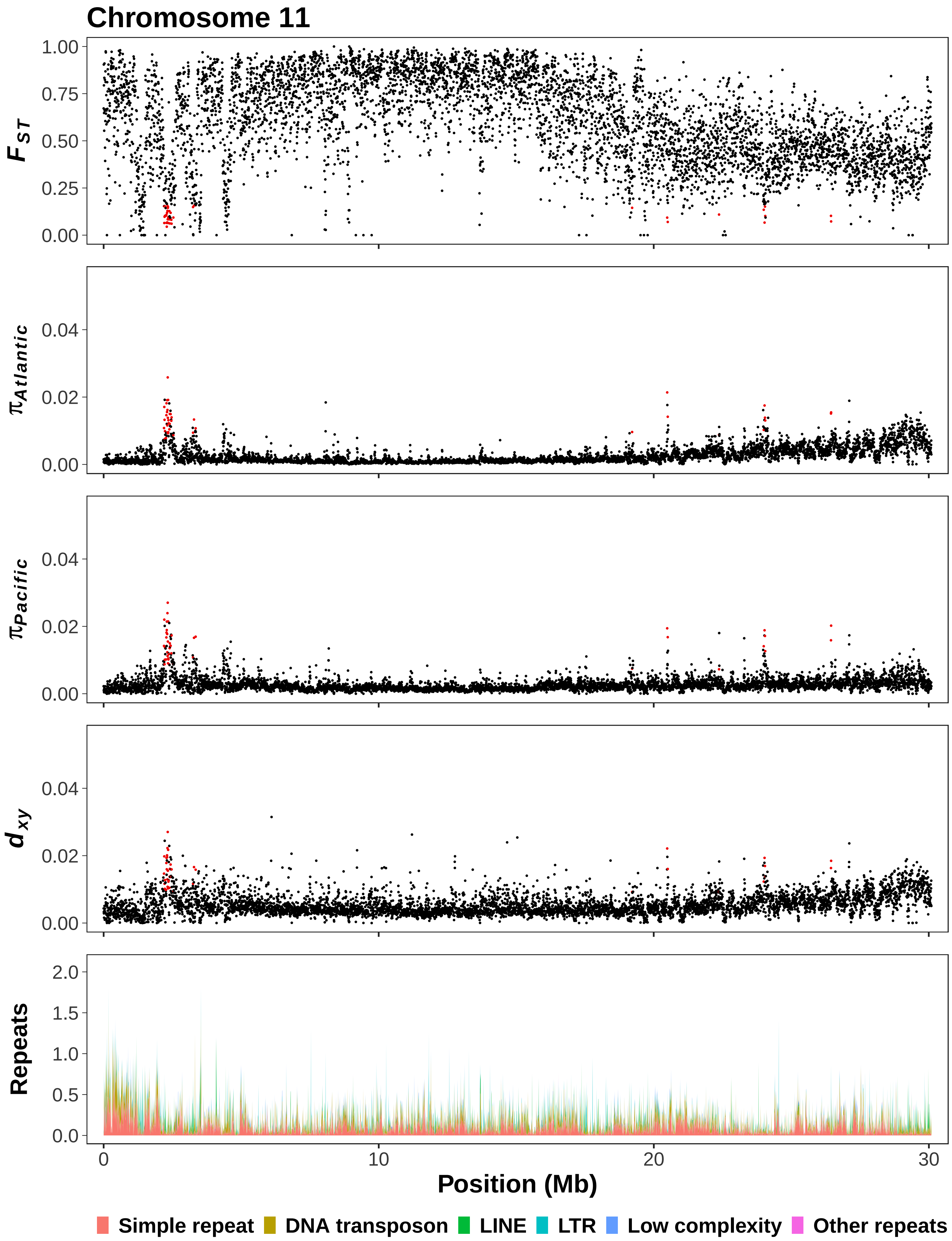

Chromosome 11
F_{ST}
1.00
0.75
0.50
0.25
0.00
$\pi_{Atlantic}$
0.04
0.02
0.00
$\pi_{Pacific}$
0.04
0.02
0.00
d_{xy}
0.04
0.02
0.00
Repeats
2.0
1.5
1.0
0.5
0.0
0
10
20
30
Position (Mb)
Simple repeat
DNA transposon
LINE
LTR
Low complexity
Other repeats

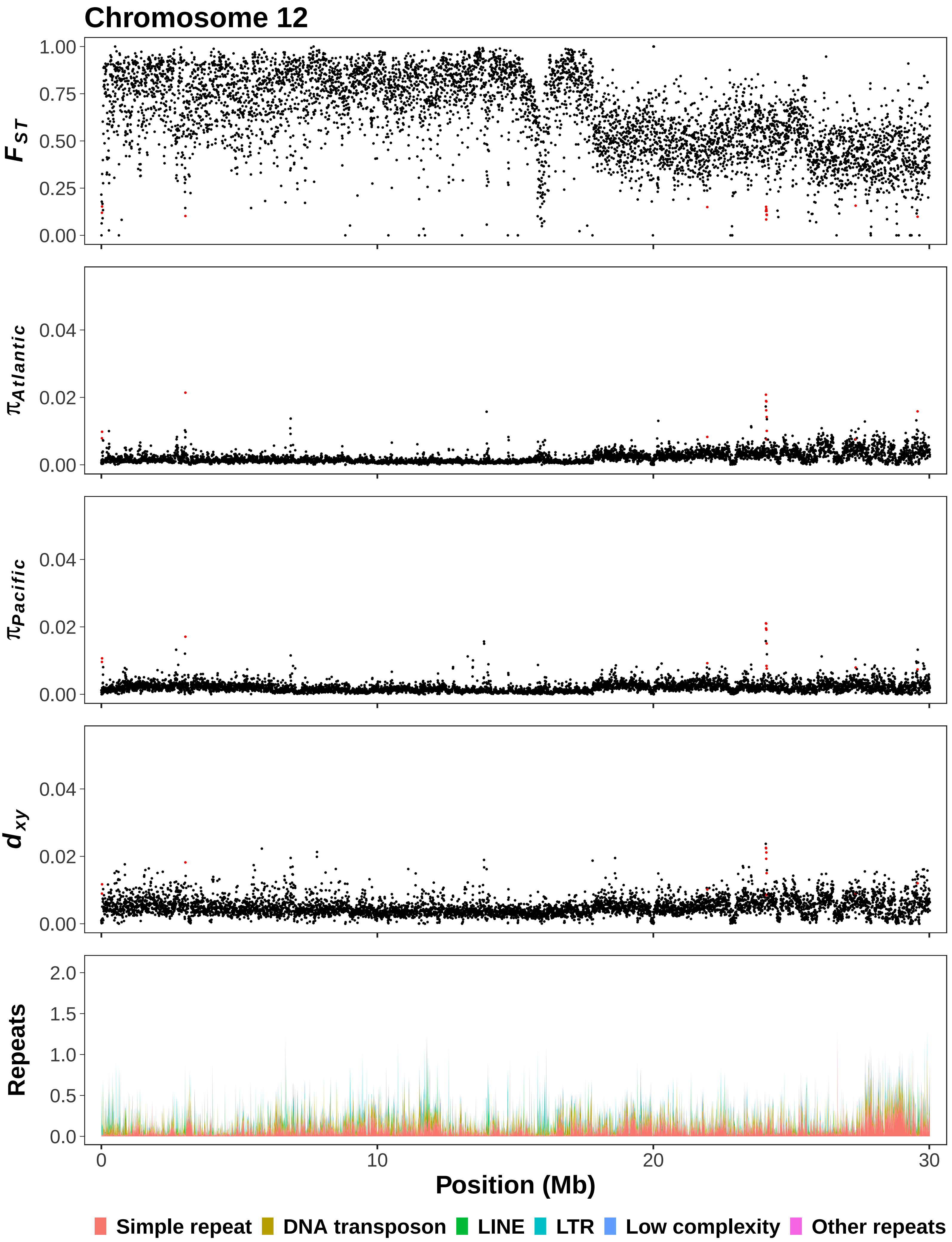

Chromosome 12
F_{ST}
1.00
0.75
0.50
0.25
0.00
$\pi_{Atlantic}$
0.04
0.02
0.00
$\pi_{Pacific}$
0.04
0.02
0.00
d_{xy}
0.04
0.02
0.00
Repeats
2.0
1.5
1.0
0.5
0.0
0
10
20
30
Position (Mb)
Simple repeat
DNA transposon
LINE
LTR
Low complexity
Other repeats

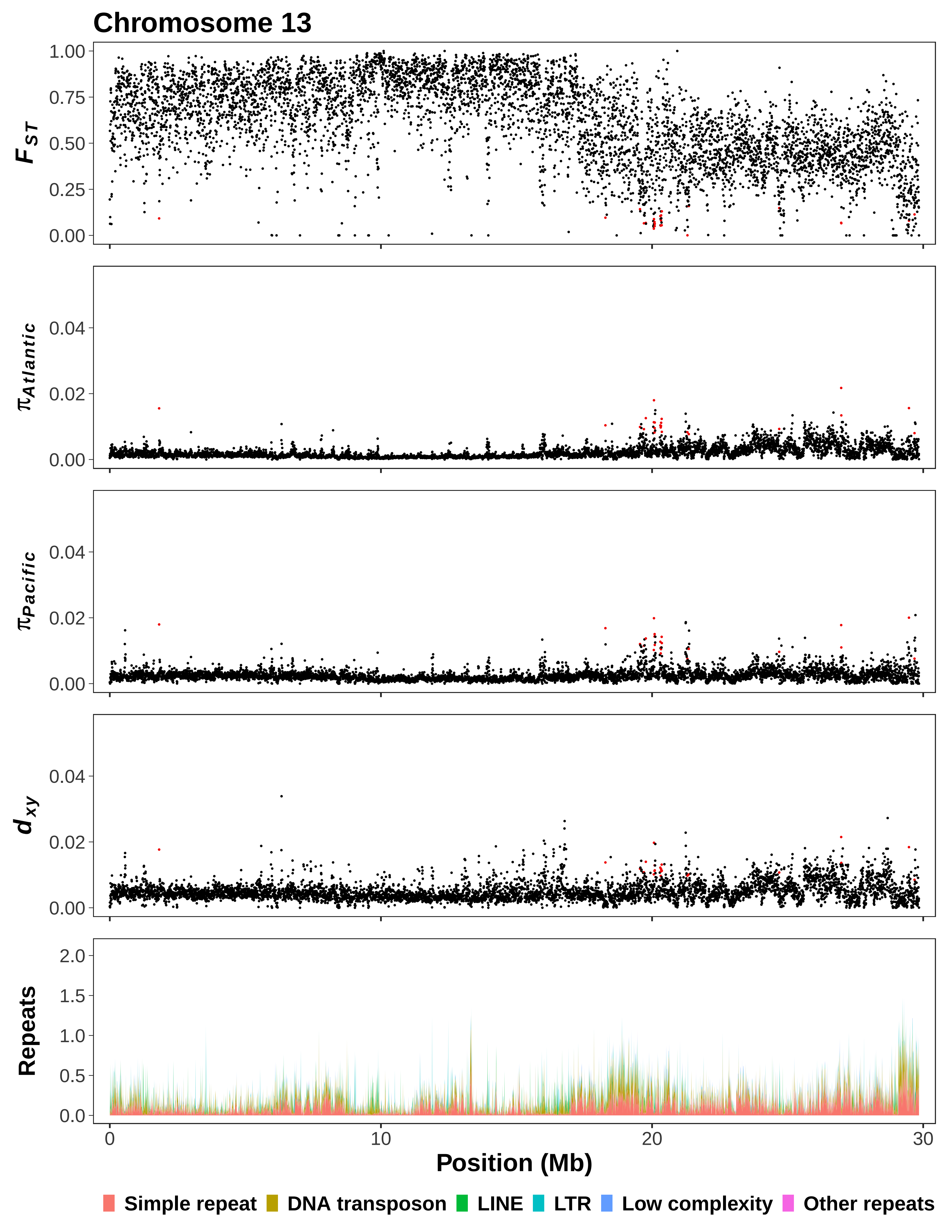

Chromosome 13
F_{ST}
1.00
0.75
0.50
0.25
0.00
$\pi_{Atlantic}$
0.04
0.02
0.00
$\pi_{Pacific}$
0.04
0.02
0.00
d_{xy}
0.04
0.02
0.00
Repeats
2.0
1.5
1.0
0.5
0.0
0
10
20
30
Position (Mb)
Simple repeat
DNA transposon
LINE
LTR
Low complexity
Other repeats

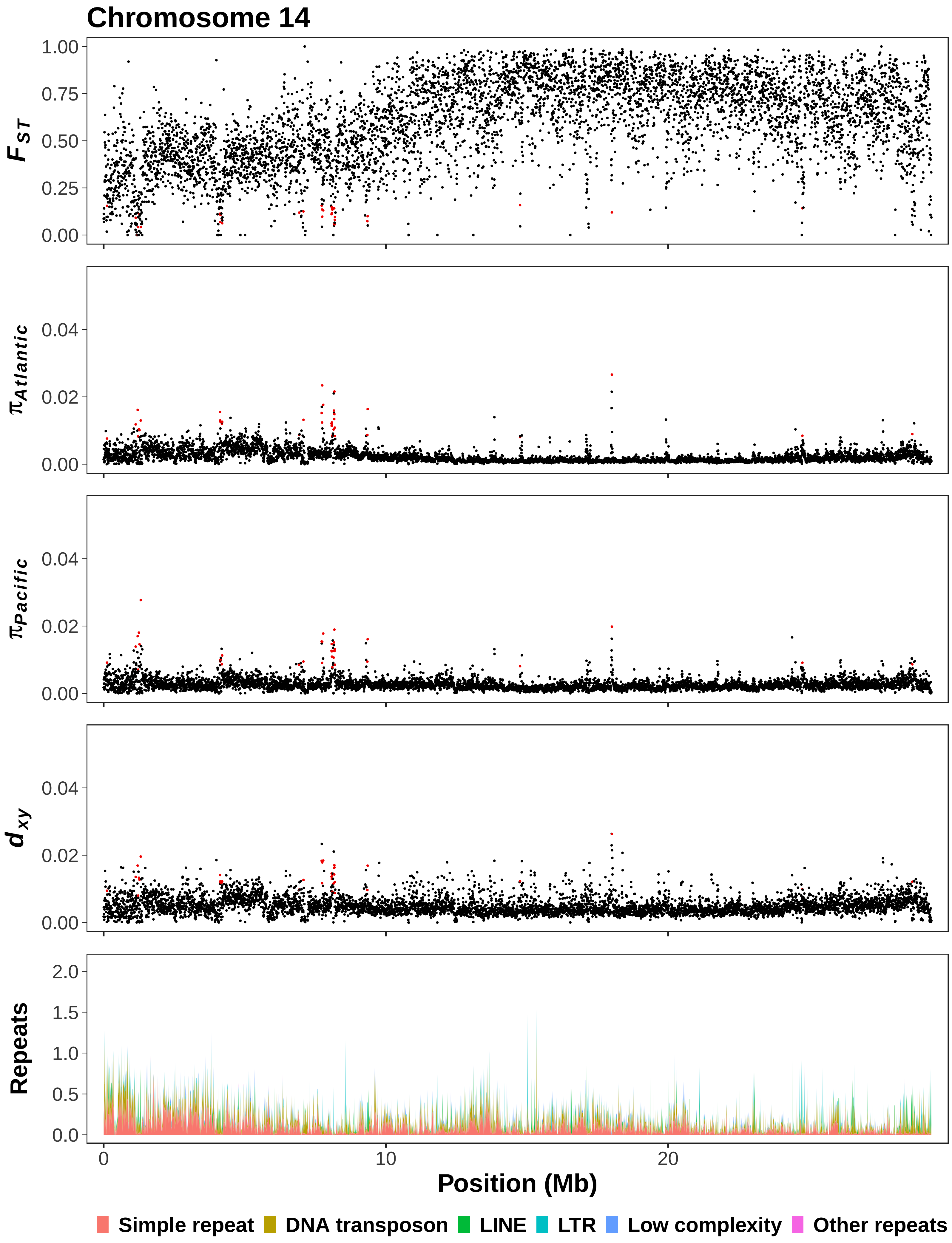

Chromosome 14
F_ST
π_Atlantic
π_Pacific
d_xy
Repeats
Position (Mb)
0
10
20
1.00
0.75
0.50
0.25
0.00
0.04
0.02
2.0
1.5
1.0
0.5
0.0
Simple repeat
DNA transposon
LINE
LTR
Low complexity
Other repeats

Chromosome 15

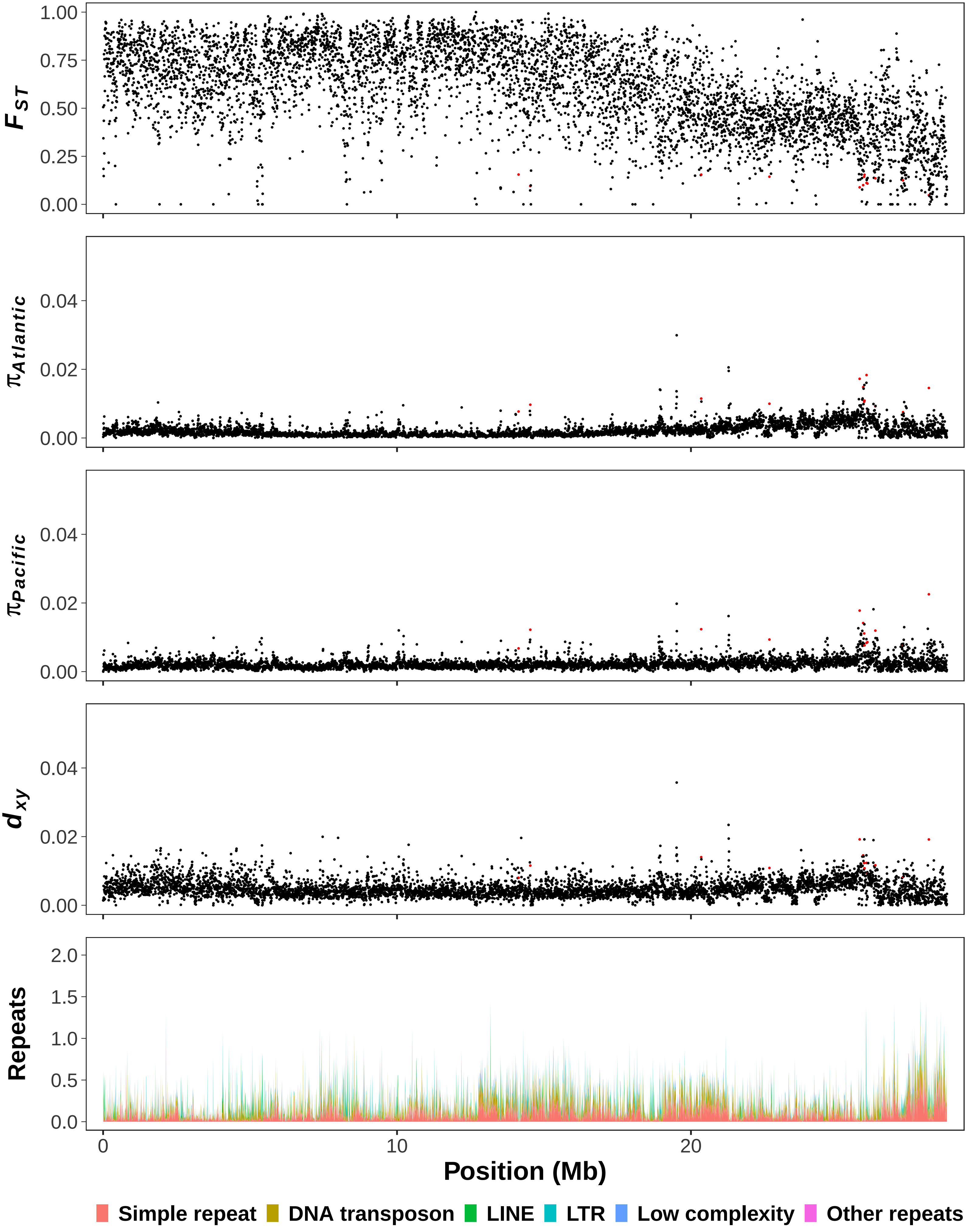

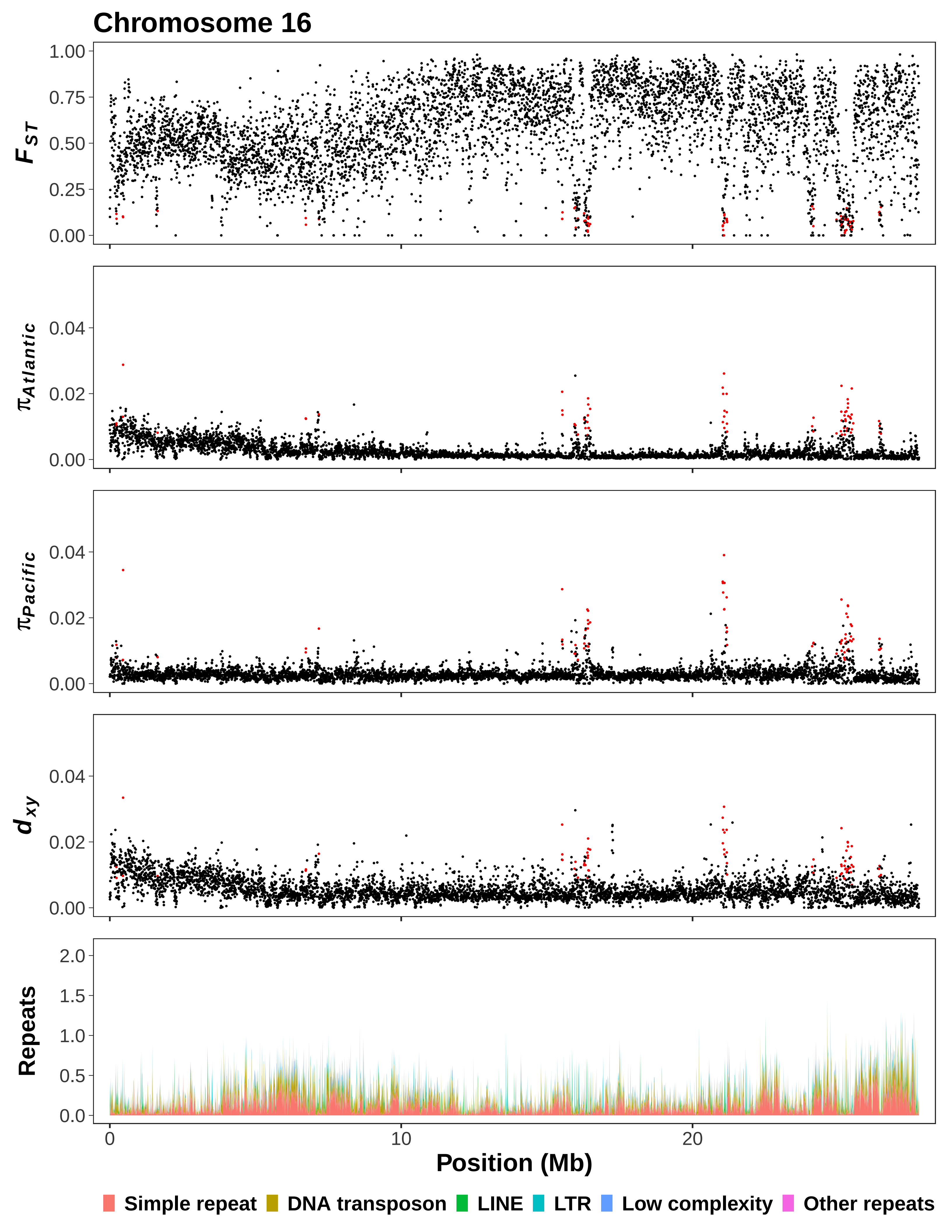

Chromosome 16
F_{ST}
1.00
0.75
0.50
0.25
0.00
$\pi_{Atlantic}$
0.04
0.02
0.00
$\pi_{Pacific}$
0.04
0.02
0.00
d_{xy}
0.04
0.02
0.00
Repeats
2.0
1.5
1.0
0.5
0.0
0
10
20
Position (Mb)
Simple repeat
DNA transposon
LINE
LTR
Low complexity
Other repeats

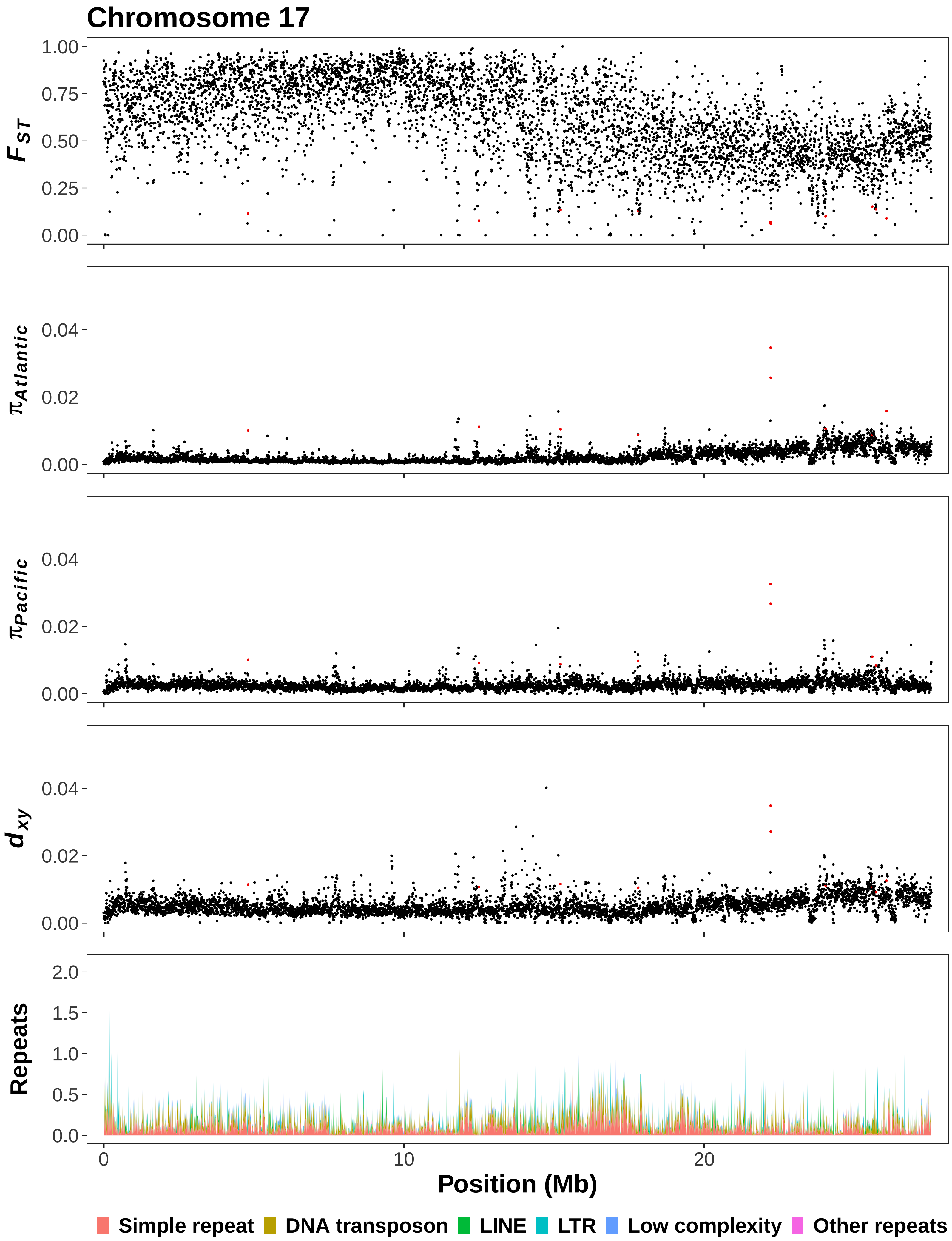
Chromosome 17
F_{ST}
1.00
0.75
0.50
0.25
0.00
$\pi_{Atlantic}$
0.04
0.02
0.00
$\pi_{Pacific}$
0.04
0.02
0.00
d_{xy}
0.04
0.02
0.00
Repeats
2.0
1.5
1.0
0.5
0.0
0
10
20
Position (Mb)
Simple repeat
DNA transposon
LINE
LTR
Low complexity
Other repeats

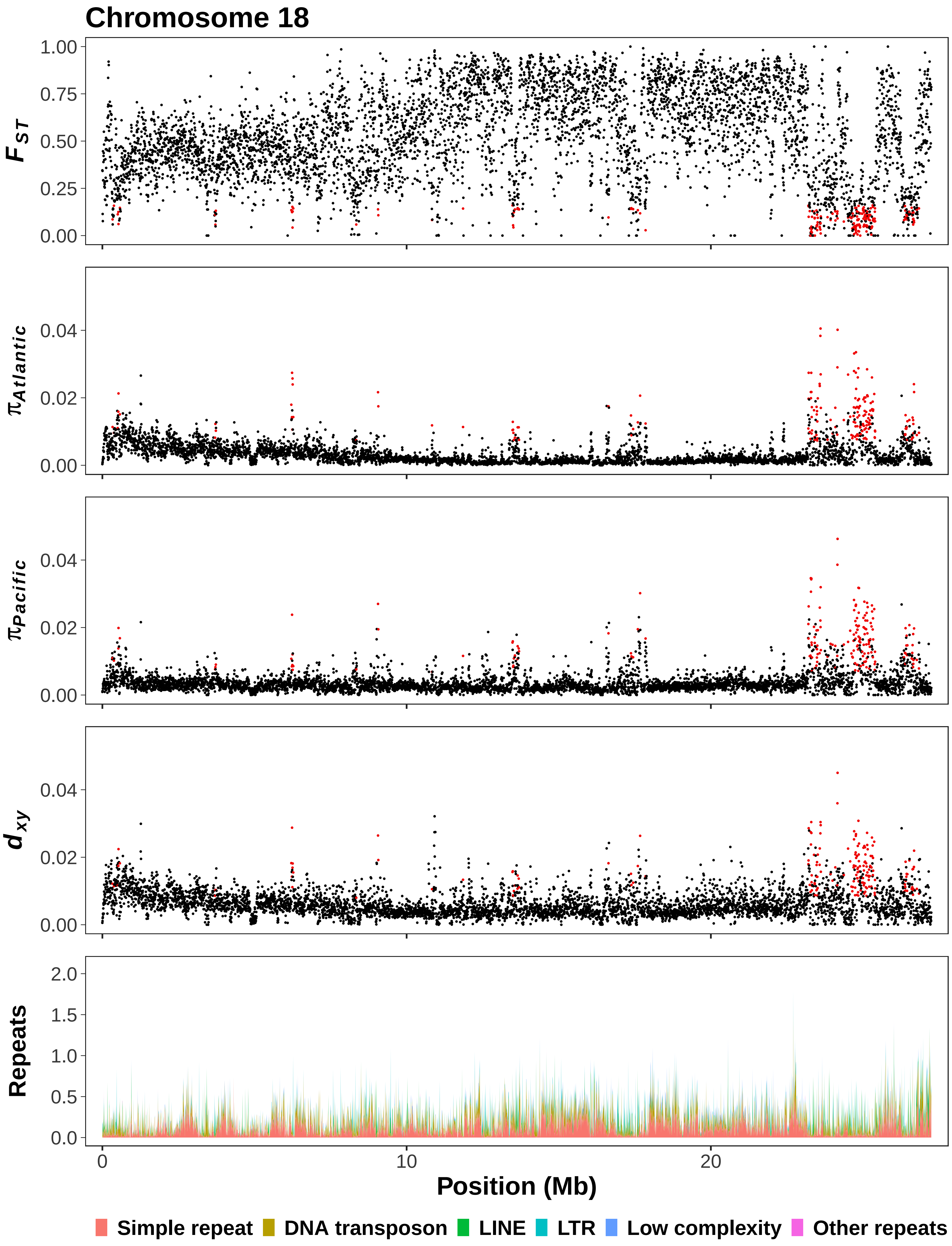
Chromosome 18
F_{ST}
1.00
0.75
0.50
0.25
0.00
$\pi_{Atlantic}$
0.04
0.02
0.00
$\pi_{Pacific}$
0.04
0.02
0.00
d_{xy}
0.04
0.02
0.00
Repeats
2.0
1.5
1.0
0.5
0.0
0
10
20
Position (Mb)
Simple repeat
DNA transposon
LINE
LTR
Low complexity
Other repeats

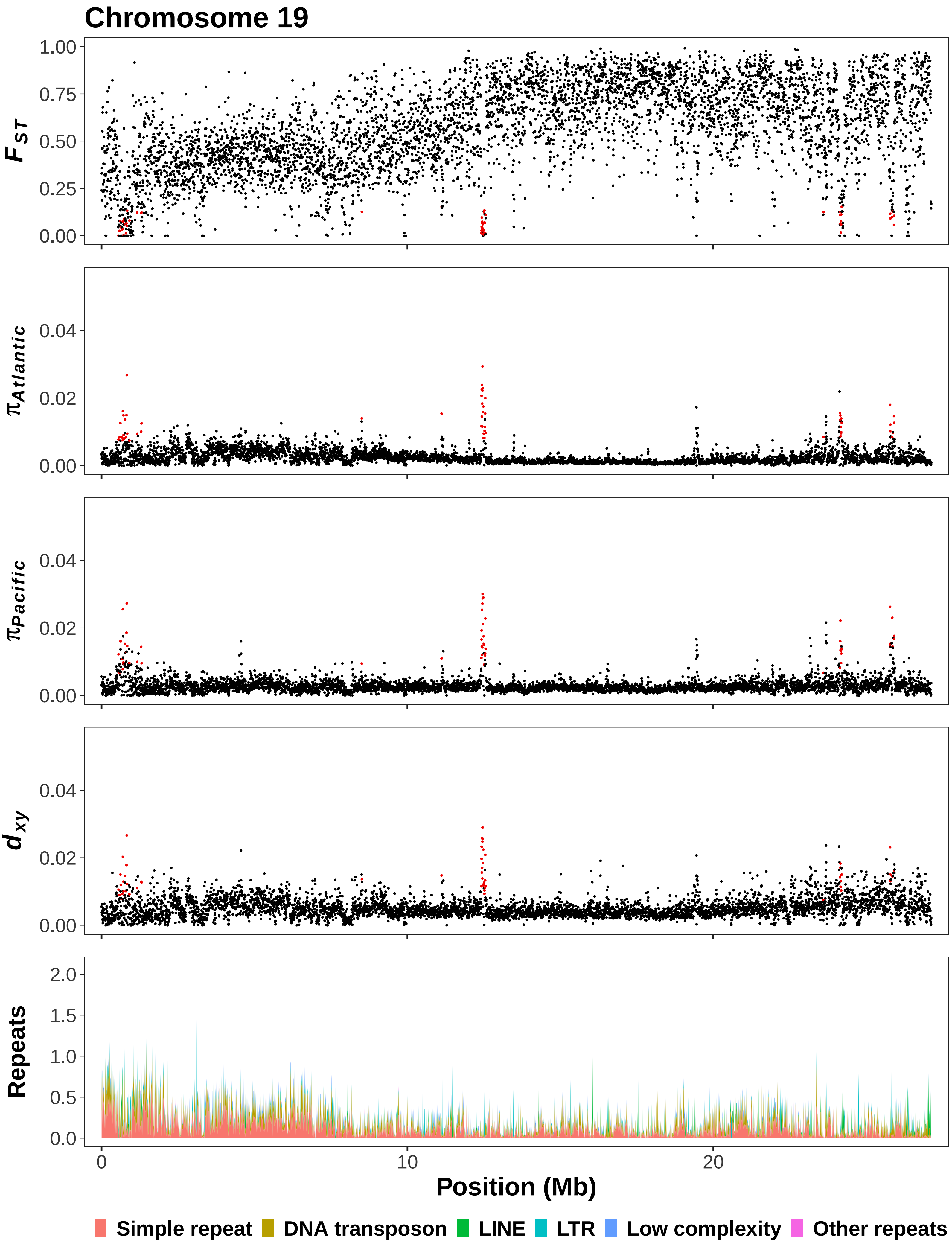

Chromosome 19
F_{ST}
1.00
0.75
0.50
0.25
0.00
$\pi_{Atlantic}$
0.04
0.02
0.00
$\pi_{Pacific}$
0.04
0.02
0.00
d_{xy}
0.04
0.02
0.00
Repeats
2.0
1.5
1.0
0.5
0.0
0
10
20
Position (Mb)
Simple repeat
DNA transposon
LINE
LTR
Low complexity
Other repeats

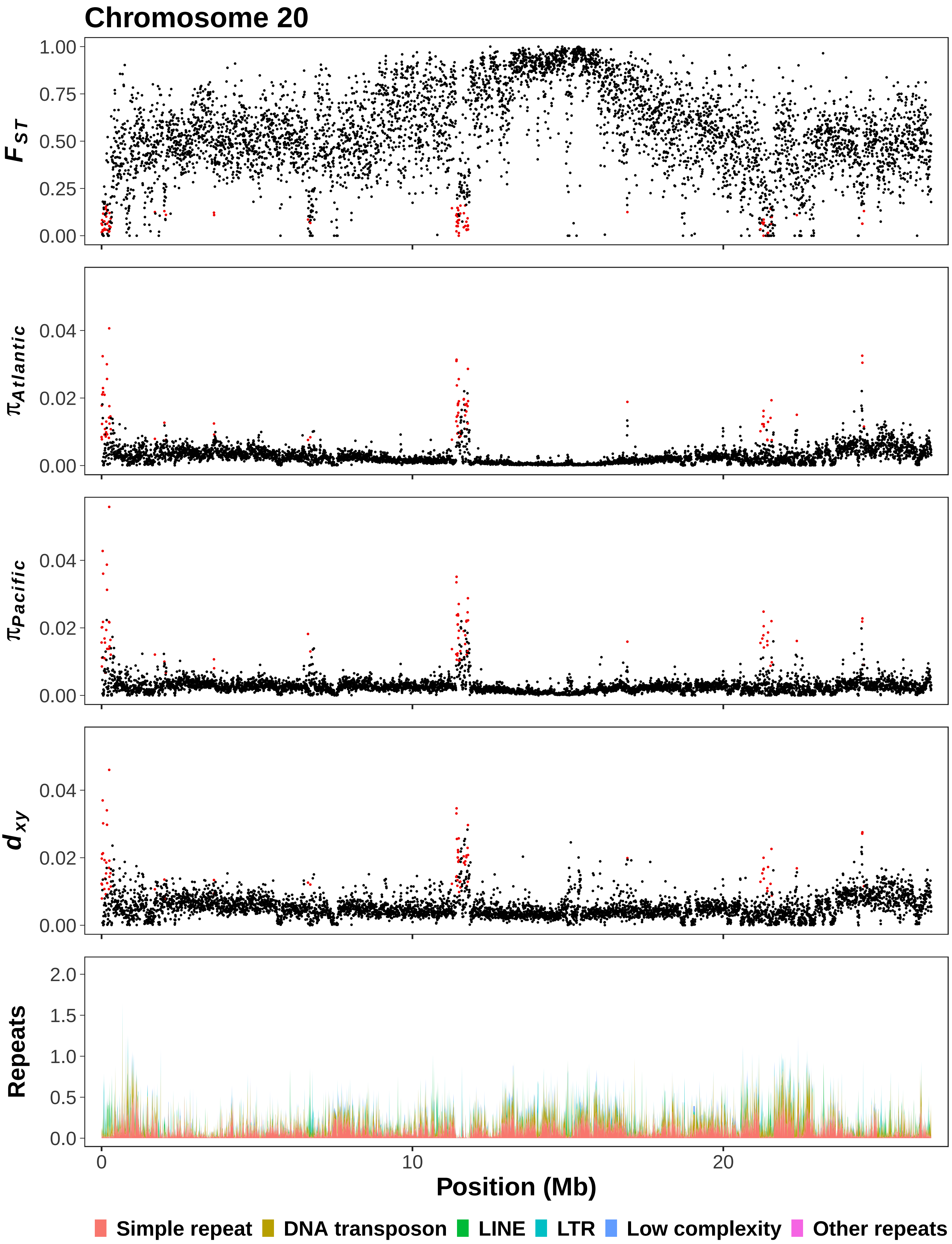

Chromosome 20
F_{ST}
1.00
0.75
0.50
0.25
0.00
$\pi_{Atlantic}$
0.04
0.02
0.00
$\pi_{Pacific}$
0.04
0.02
0.00
d_{xy}
0.04
0.02
0.00
Repeats
2.0
1.5
1.0
0.5
0.0
0
10
20
Position (Mb)
Simple repeat
DNA transposon
LINE
LTR
Low complexity
Other repeats

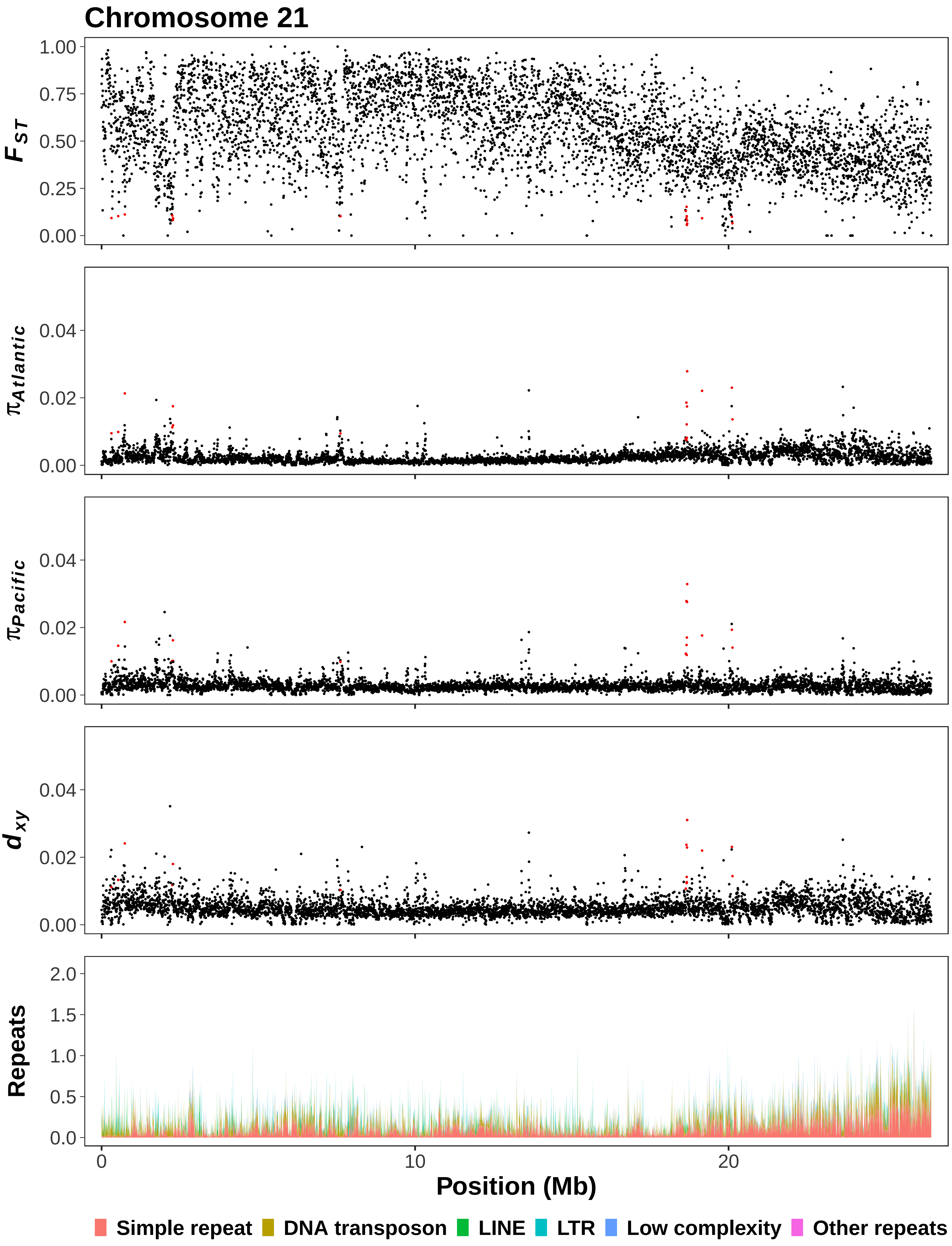

Chromosome 21
F_ST
1.00
0.75
0.50
0.25
0.00
π_Atlantic
0.04
0.02
0.00
π_Pacific
0.04
0.02
0.00
d_xy
0.04
0.02
0.00
Repeats
2.0
1.5
1.0
0.5
0.0
0
10
20
Position (Mb)
Simple repeat
DNA transposon
LINE
LTR
Low complexity
Other repeats

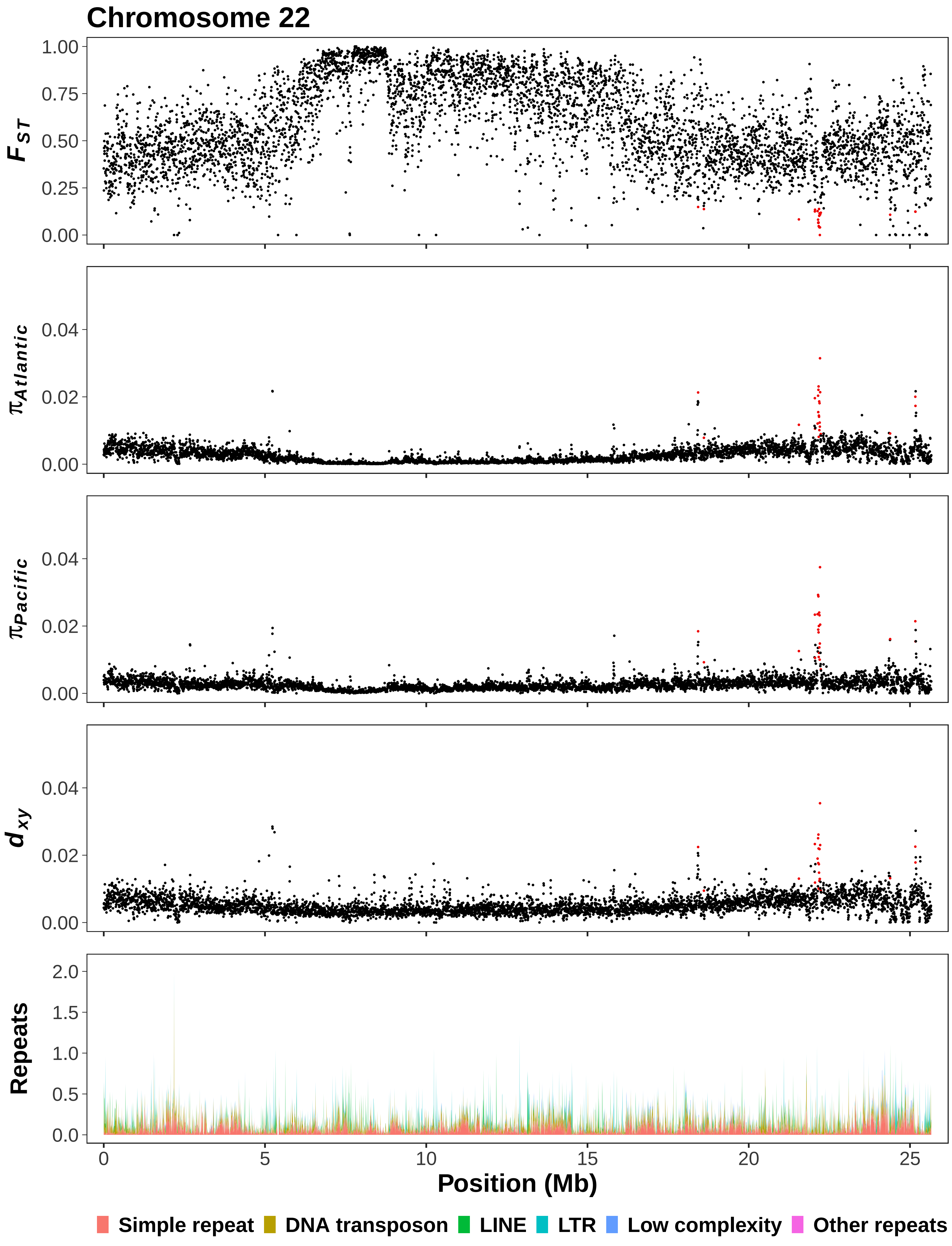

Chromosome 22
F_{ST}
1.00
0.75
0.50
0.25
0.00
$\pi_{Atlantic}$
0.04
0.02
0.00
$\pi_{Pacific}$
0.04
0.02
0.00
d_{xy}
0.04
0.02
0.00
Repeats
2.0
1.5
1.0
0.5
0.0
0
5
10
15
20
25
Position (Mb)
Simple repeat
DNA transposon
LINE
LTR
Low complexity
Other repeats

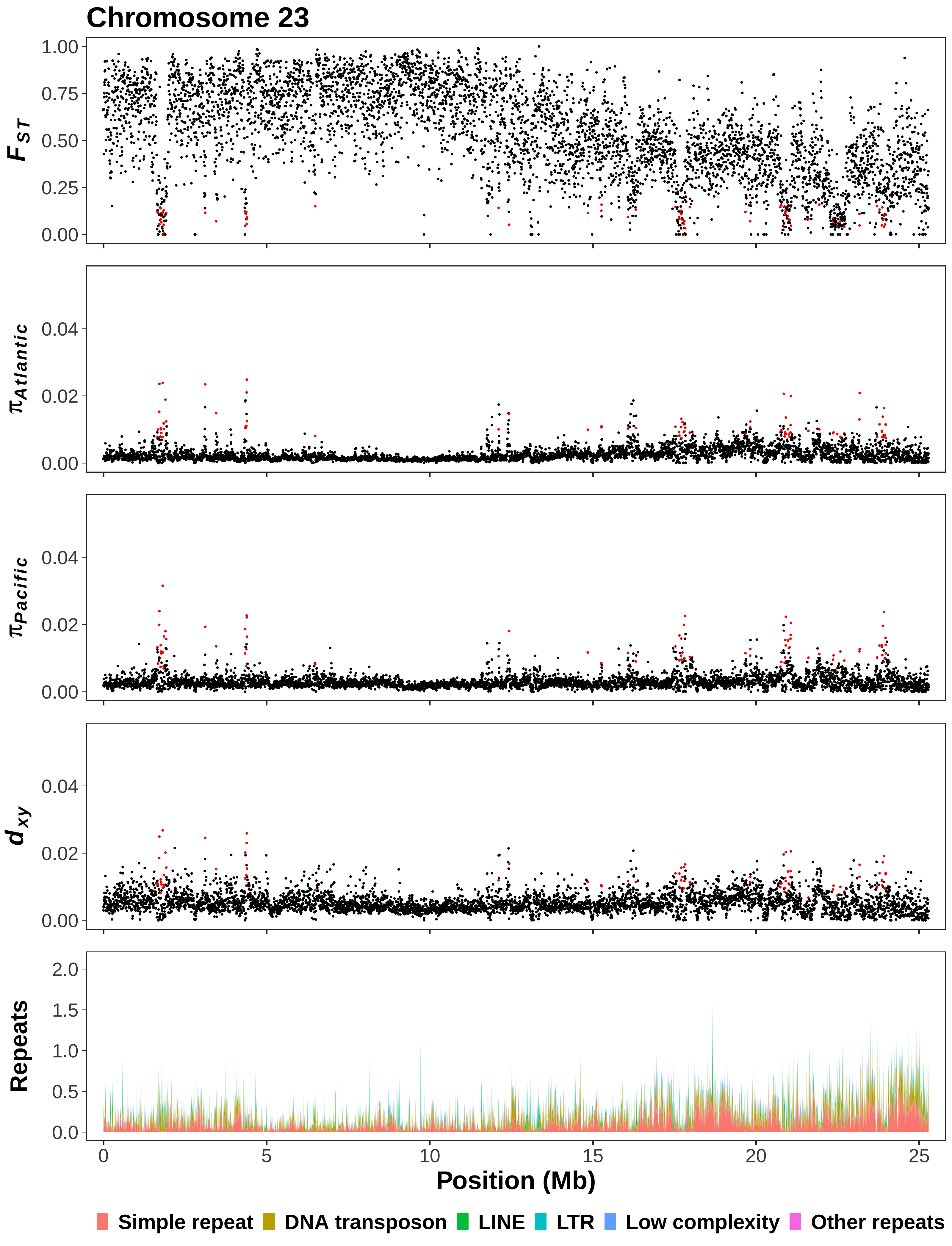

Chromosome 23
F_{ST}
1.00
0.75
0.50
0.25
0.00
$\pi_{Atlantic}$
$\pi_{Pacific}$
d_{xy}
0.04
0.02
0.00
Repeats
2.0
1.5
1.0
0.5
0.0
0
5
10
15
20
25
Position (Mb)
Simple repeat
DNA transposon
LINE
LTR
Low complexity
Other repeats

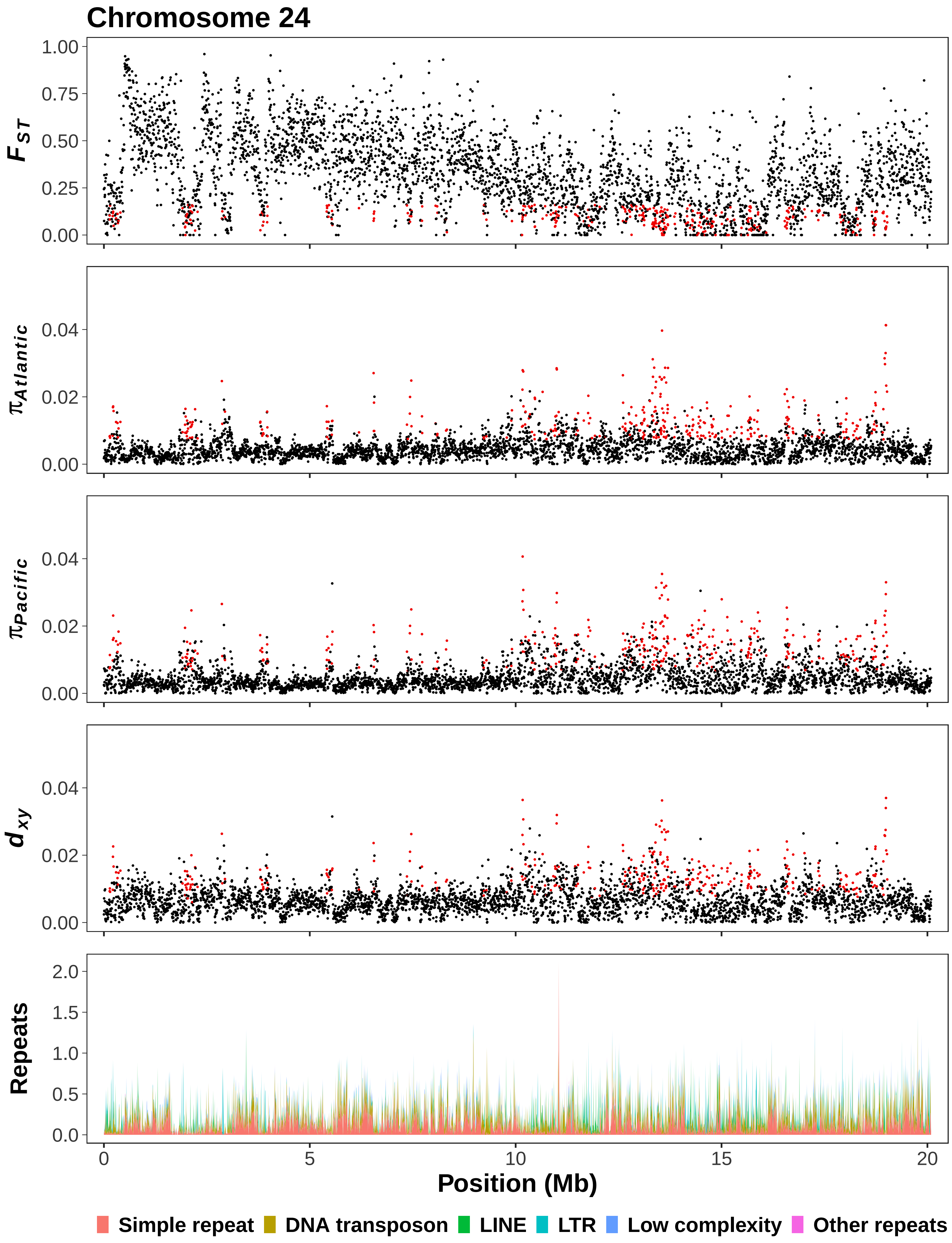
Chromosome 24
F_{ST}
1.00
0.75
0.50
0.25
0.00
$\pi_{Atlantic}$
0.04
0.02
0.00
$\pi_{Pacific}$
0.04
0.02
0.00
d_{xy}
0.04
0.02
0.00
Repeats
2.0
1.5
1.0
0.5
0.0
0
5
10
15
20
Position (Mb)
Simple repeat
DNA transposon
LINE
LTR
Low complexity
Other repeats

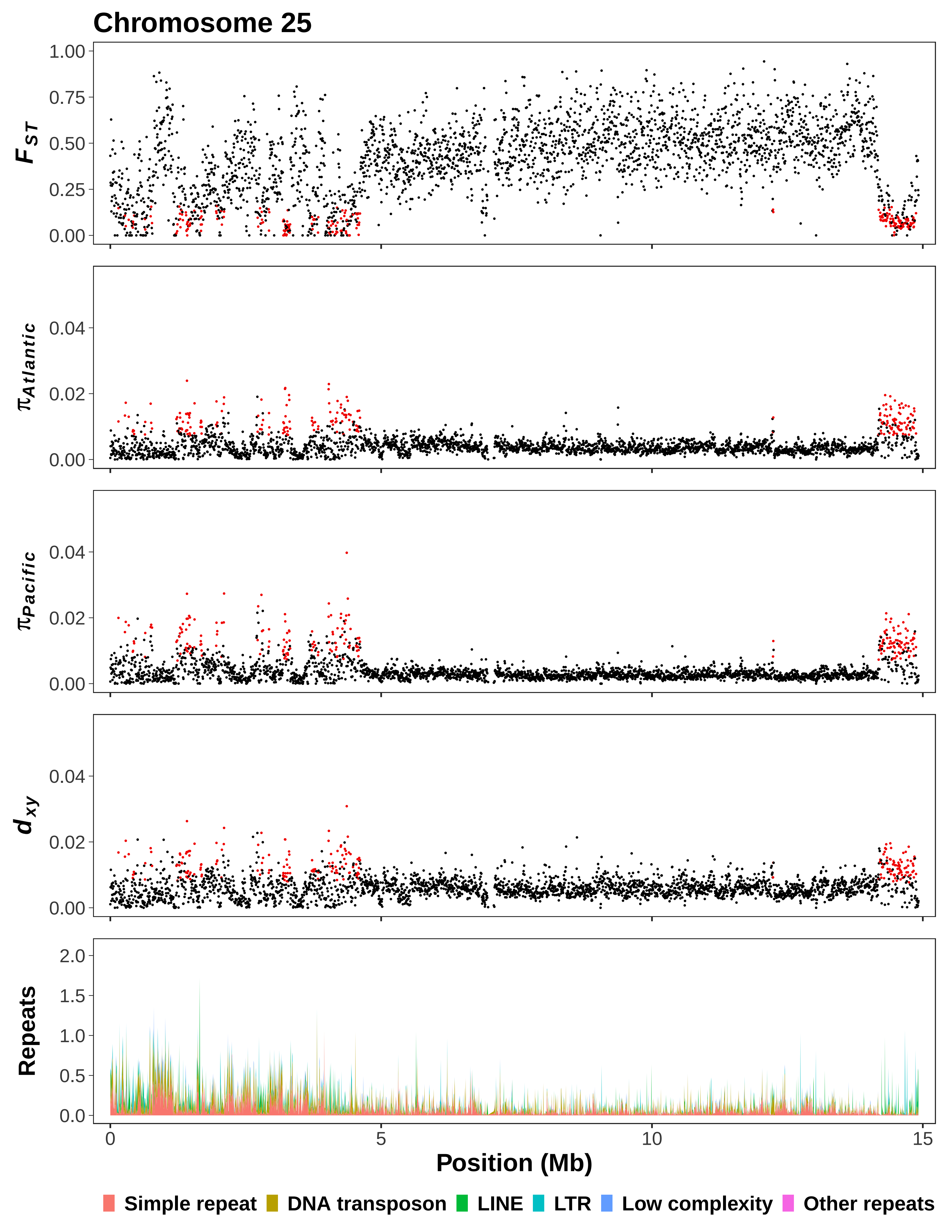
Chromosome 25
F_{ST}
$\pi_{Atlantic}$
$\pi_{Pacific}$
d_{xy}
Repeats
Position (Mb)
Simple repeat
DNA transposon
LINE
LTR
Low complexity
Other repeats

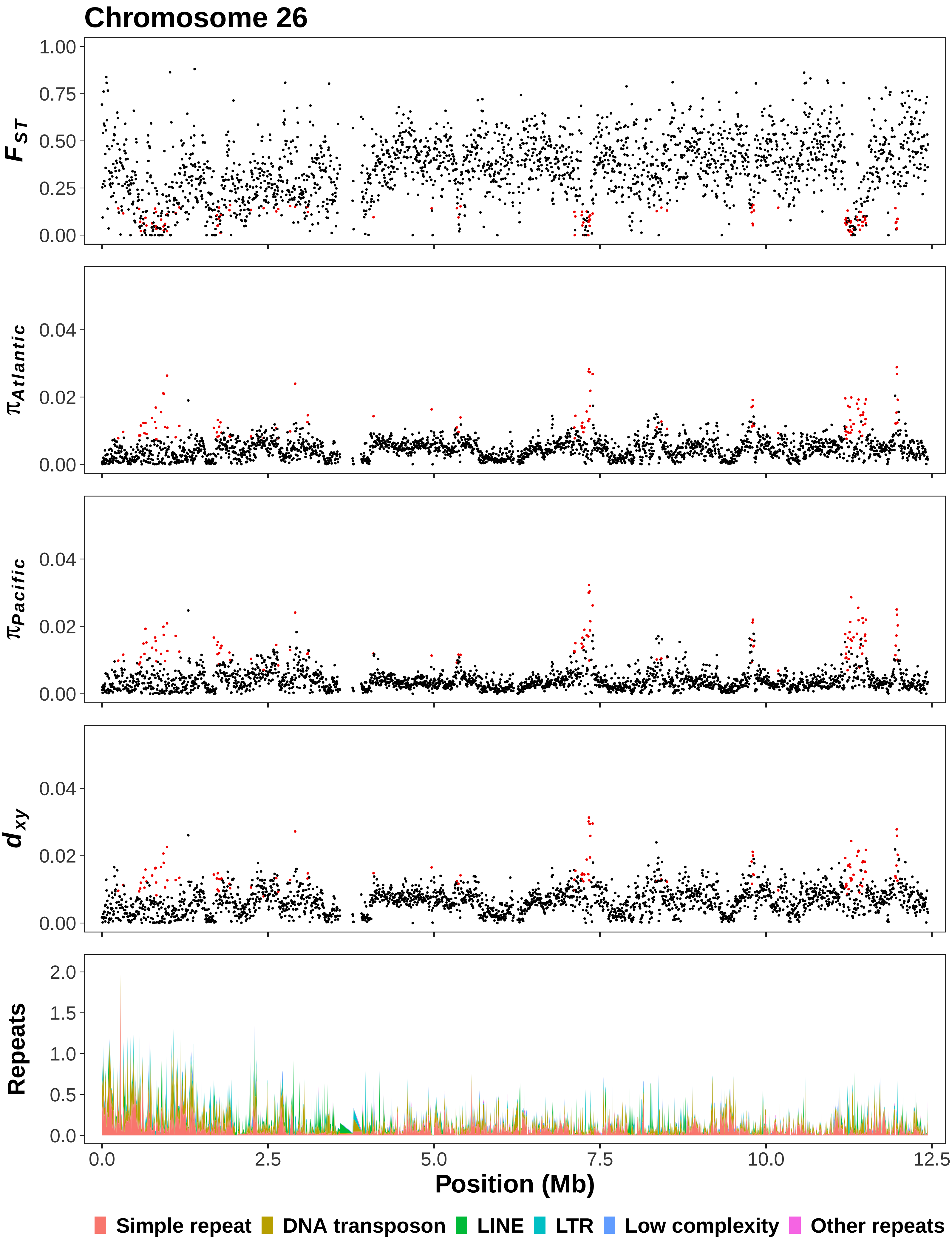
Chromosome 26
F_ST
π_Atlantic
π_Pacific
d_xy
Repeats
1.00
0.75
0.50
0.25
0.00
0.04
0.02
0.00
2.0
1.5
1.0
0.5
0.0
2.5
5.0
7.5
10.0
12.5
Position (Mb)
Simple repeat
DNA transposon
LINE
LTR
Low complexity
Other repeats
