# Supplementary material for: Copy number variation and elevated genetic diversity at immune trait loci in Atlantic and Pacific herring
Source: BMC Genomics. 2024 May 10;25:459. doi: 10.1186/s12864-024-10380-5 (PMC11088111; doi:10.1186/s12864-024-10380-5)
Supplement: Supplementary file 3 — Supplementary Material 3: Additional file 3: Fig. S2. [file 12864_2024_10380_MOESM3_ESM.pdf]

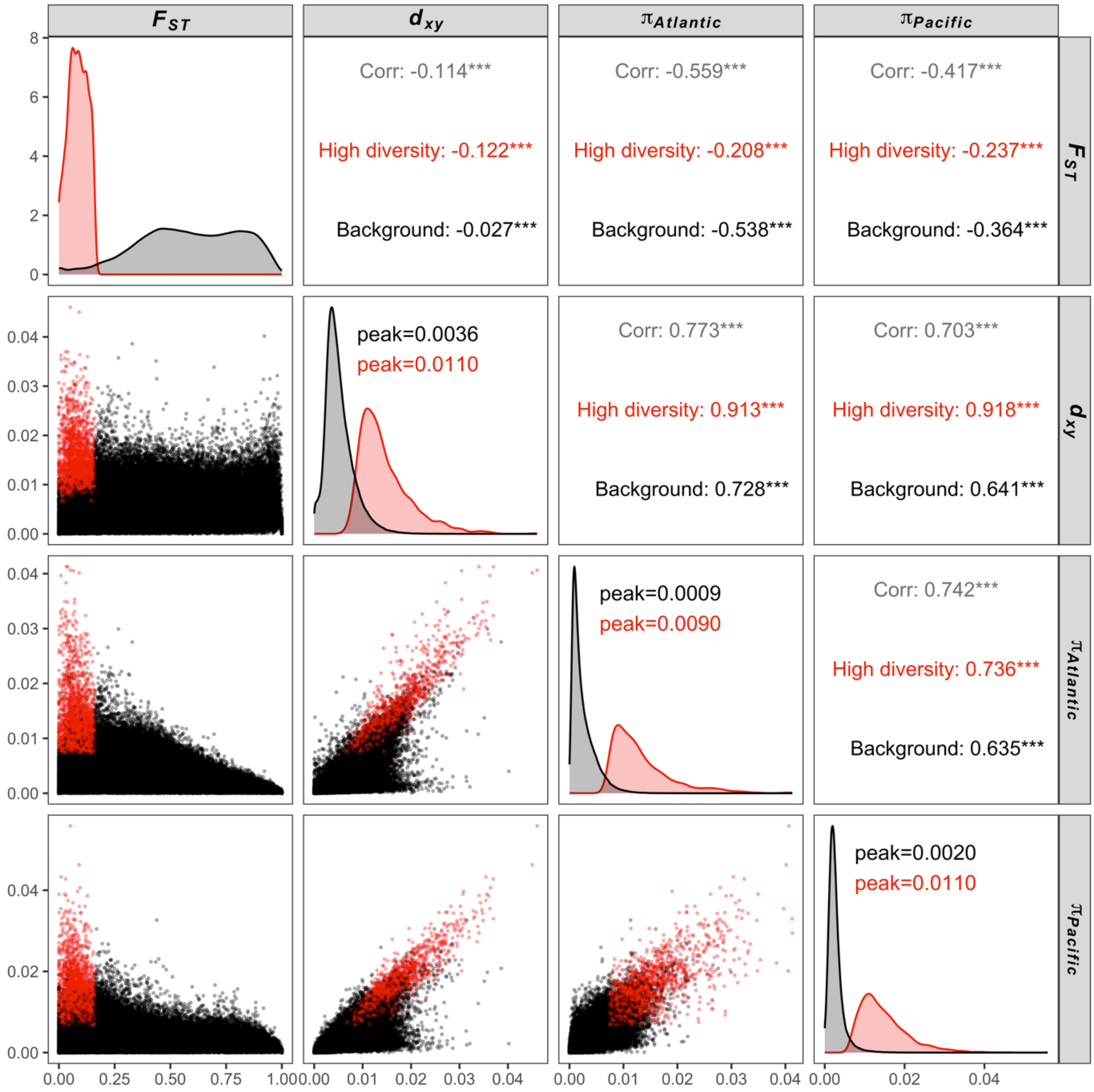

Figure S2. Correlation matrix for population genetic diversity parameters between Atlantic and Pacific herring estimated within nonoverlapping 5 kb windows. The diagonal shows the density distribution of parameters (the areas under the two density curves in each block are not proportional to the number of bins in each group), the lower triangle displays joint distributions and upper triangle summarises Pearson correlation coefficients between pairs of parameters within the high-diversity (red) and background (black) windows.
