# Supplementary material for: Copy number variation and elevated genetic diversity at immune trait loci in Atlantic and Pacific herring
Source: BMC Genomics. 2024 May 10;25:459. doi: 10.1186/s12864-024-10380-5 (PMC11088111; doi:10.1186/s12864-024-10380-5)
Supplement: Supplementary file 4 — Supplementary Material 4: Additional file 4: Fig. S3. [file 12864_2024_10380_MOESM4_ESM.pdf]

A) Biological Process

1: homophilic cell adhesion via plasma membrane adhesion molecules
2: immune response
3: phagocytosis, recognition
4: complement activation, classical pathway
5: positive regulation of B cell activation
6: phagocytosis, engulfment
7: regulation of apoptotic process
8: B cell receptor signaling pathway
9: defense response to bacterium
10: innate immune response
11: defense response to Gram-negative bacterium
12: defense response to Gram-positive bacterium
13: negative regulation of alpha-beta T cell proliferation
14: proteasomal ubiquitin-independent protein catabolic process

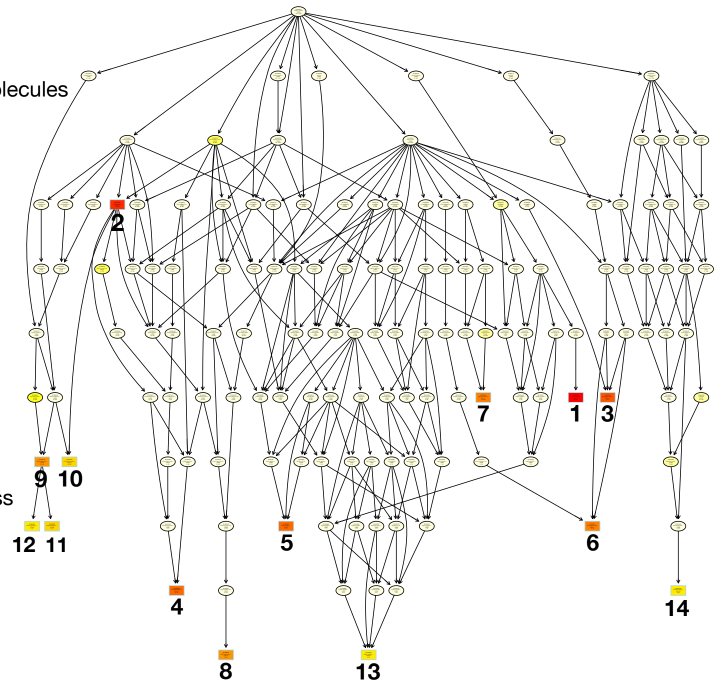

B) Molecular Function

1: GTP binding
2: immunoglobulin receptor binding
3: antigen binding
4: threonine-type endopeptidase activity
5: zinc ion binding

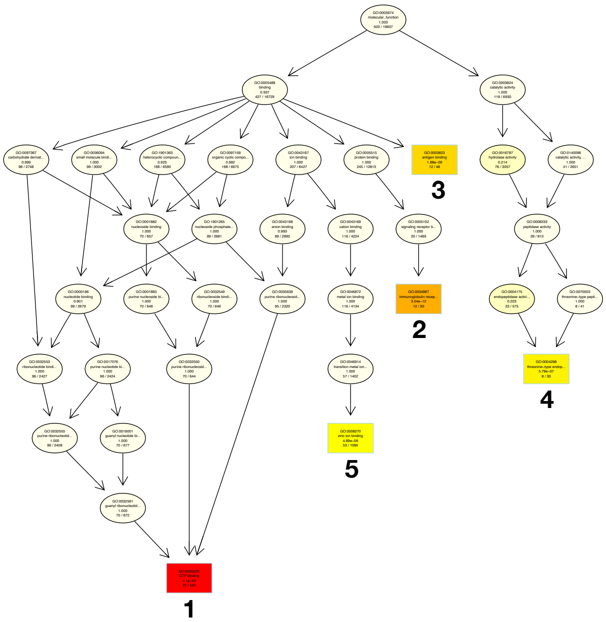

C) Cellular component

1: intracellular anatomical structure
2: MHC class II protein complex
3: immunoglobulin complex, circulating
4: external side of plasma membrane
5: nucleosome
6: proteasome core complex

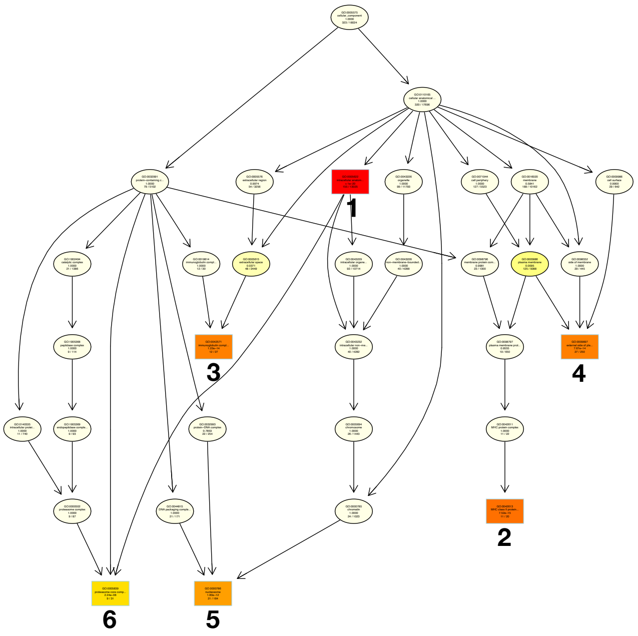

Figure S3. Gene ontology subgraphs induced by significant terms in gene set enrichment analysis. Rectangles represent significant biological process (**A**), molecular function (**B**) and cellular component (**C**) terms ($P \leq 0.01$; corrected for multiple testing with the Bonferroni method). The most significant nodes are shown with dark red and light yellow represents the least significant nodes.
