# Supplementary material for: Copy number variation and elevated genetic diversity at immune trait loci in Atlantic and Pacific herring
Source: BMC Genomics. 2024 May 10;25:459. doi: 10.1186/s12864-024-10380-5 (PMC11088111; doi:10.1186/s12864-024-10380-5)
Supplement: Supplementary file 6 — Supplementary Material 6: Additional file 6: Fig. S4. [file 12864_2024_10380_MOESM6_ESM.pdf]

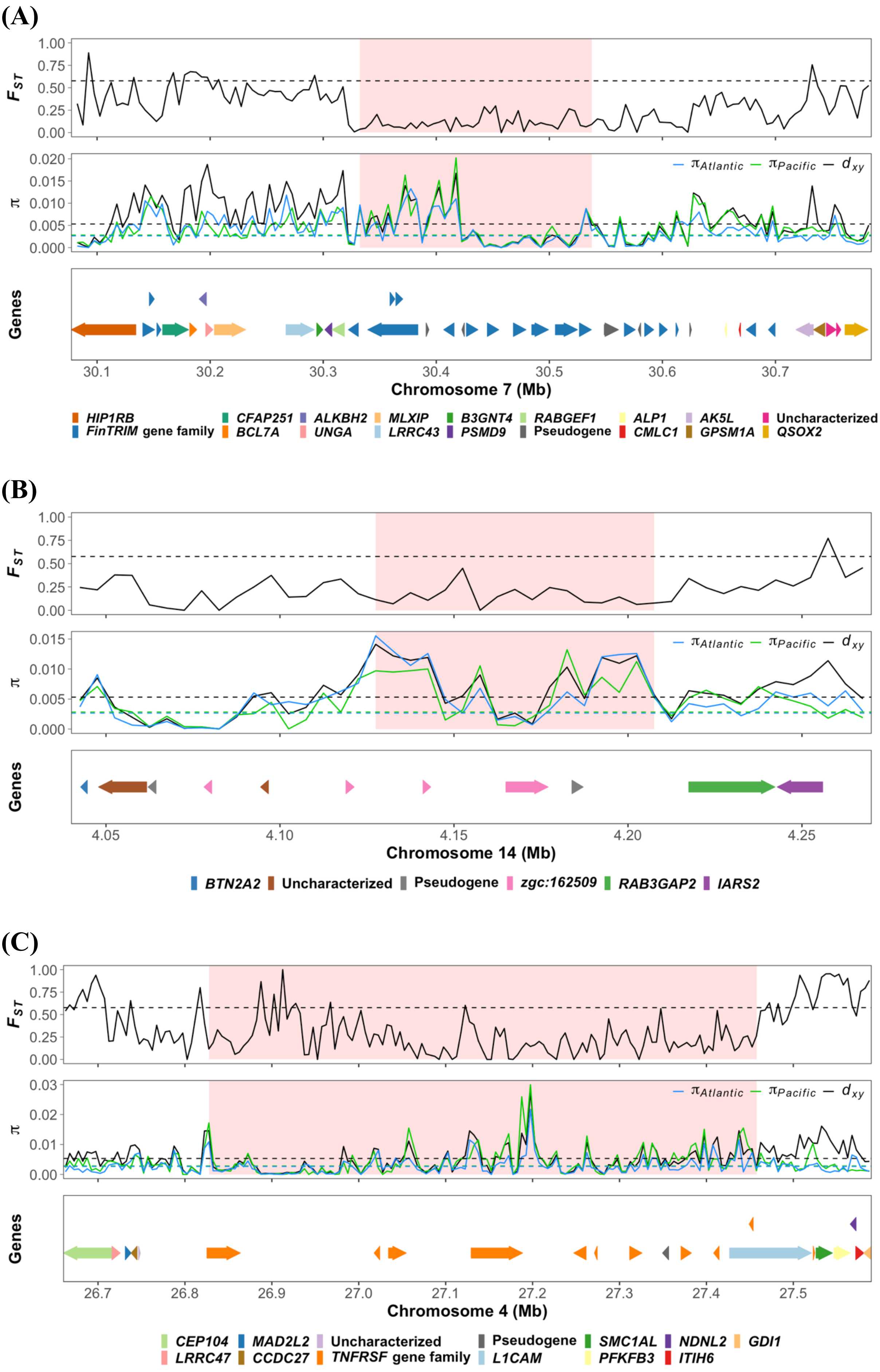
(A)
F_ST
π
πAtlantic
πPacific
d_xy
Genes
Chromosome 7 (Mb)
HIP1RB
FinTRIM gene family
CFAP251
BCL7A
ALKBH2
UNGA
MLXIP
LRRC43
B3GNT4
PSMD9
RABGEF1
Pseudogene
ALP1
CMLC1
AK5L
GPSM1A
Uncharacterized
QSOX2
(B)
F_ST
π
πAtlantic
πPacific
d_xy
Genes
Chromosome 14 (Mb)
BTN2A2
Uncharacterized
Pseudogene
zgc:162509
RAB3GAP2
IARS2
(C)
F_ST
π
πAtlantic
πPacific
d_xy
Genes
Chromosome 4 (Mb)
CEP104
LRRC47
MAD2L2
CCDC27
Uncharacterized
TNFRSF gene family
Pseudogene
L1CAM
SMC1AL
PFKFB3
NDNL2
ITIH6
GDI1

Figure S4. Illustrative examples of immune-related gene family clusters in the high-diversity regions detected in Atlantic vs. Pacific herring population comparison. The colored area in F_{ST} and π plots represent merged high-diversity regions in each example. Dashed lines depict respective genome-wide averages. The bottom track in each figure illustrates gene organization in the Atlantic herring reference genome (annotation source: NCBI Clupea harengus Annotation Release 102), indicating a cluster of *FinTRIM* (**A**), *zgc:162509* (**B**) and *TNFRSF* genes (**C**). On the upper row are the nested genes and partially overlapping genes. Color code for genes is given below each figure.
