# Supplementary material for: Copy number variation and elevated genetic diversity at immune trait loci in Atlantic and Pacific herring
Source: BMC Genomics. 2024 May 10;25:459. doi: 10.1186/s12864-024-10380-5 (PMC11088111; doi:10.1186/s12864-024-10380-5)
Supplement: Supplementary file 8 — Supplementary Material 8: Additional file 8: Table S4. [file 12864_2024_10380_MOESM8_ESM.pdf]

Table S4. Examples of gene clusters at regions exhibiting low differentiation and high nucleotide diversity in other fishes. This table highlights gene clusters consisting of a minimum of three genes.

Species radiation	Approximate coordination	Gene cluster	Annotation source	Reference
Midas cichlid	Chr2:19350000-19500000	interferon-induced very large GTPase 1	NCBI Archocentrus centrarchus Annotation Release 100	Kautt et al. 2020
Midas cichlid	Chr2:21600000-22700000	protocadherin	NCBI Archocentrus centrarchus Annotation Release 100	Kautt et al. 2020
Midas cichlid	Chr4:17800000-19200000	Ig heavy chain variable	NCBI Archocentrus centrarchus Annotation Release 100	Kautt et al. 2020
Midas cichlid	Chr12:5900000-6600000	GTPase IMAP family member 8-like	NCBI Archocentrus centrarchus Annotation Release 100	Kautt et al. 2020
Ninespine stickleback	Chr4a:9500000-10500000	protocadherin	NCBI Pungitius pungitius Annotation Release 100	Yamasaki et al. 2020
Ninespine stickleback	Chr9:19400000-20400000	E3 ubiquitin-protein ligase TRIM39<br>carcinoembryonic antigen-related cell adhesion molecule 5	NCBI Pungitius pungitius Annotation Release 100	Yamasaki et al. 2020
Ninespine stickleback	Chr13:15800000-16300000	gastrula zinc finger protein	NCBI Pungitius pungitius Annotation Release 100	Yamasaki et al. 2020
Ninespine stickleback	Chr14:2200000-2700000	NACHT, LRR and PYD domains-containing protein 3-like<br>NLR family CARD domain-containing protein 3-like	NCBI Pungitius pungitius Annotation Release 100	Yamasaki et al. 2020
